# Reversing KDM6A phosphorylation-driven epigenetic suppression restores pyroptosis and synergizes with anti-PD-1 therapy in HNSCC

**DOI:** 10.1038/s41419-026-09196-w

**Published:** 2026-08-18

**Authors:** Yikang Ji, Xinran Zhao, Cheng Hu, Jun Chen, Zhiyin Li, Xin Hu, Mi Zhang, Qingqing Wan, Xinhua Pan, Yan Wei, Xu Wang

**Affiliations:** 1 Department of Oral and Maxillofacial-Head and Neck Oncology, Shanghai Center of Head and Neck Oncology Clinical and Translational Science, Shanghai Ninth People’s Hospital, School of Medicine, College of Stomatology, National Center for Stomatology, National Clinical Research Center for Oral Diseases, Shanghai Key Laboratory of Stomatology, Shanghai Research Institute of Stomatology, Shanghai Jiao Tong University, Shanghai, China; 2 Department of Oral Surgery, Shanghai Ninth People’s Hospital, Shanghai Jiao Tong University, School of Medicine; College of Stomatology, Shanghai Jiao Tong University; National Center for Stomatology; National Clinical Research Center for Oral Diseases; Shanghai Key Laboratory of Stomatology; Shanghai Research Institute of Stomatology, Shanghai, PR China; 3 Department of Ophthalmology, Eye & ENT Hospital, Shanghai Medical College, Fudan University, Shanghai, China; 4 NHC Key Laboratory of Myopia (Fudan University), Key Laboratory of Myopia, Chinese Academy of Medical Sciences, Shanghai Key Laboratory of Visual Impairment and Restoration (Fudan University), Shanghai, China

**Keywords:** Oral cancer, Cancer immunotherapy

## Abstract

Immune checkpoint blockade (ICB) has revolutionized head and neck squamous cell carcinoma (HNSCC) treatment. Yet, a major barrier to response is that tumor cells are epigenetically reprogrammed to resist immunogenic cell death, particularly pyroptosis. To elucidate how tumor cells are pre-conFigured for pyroptosis resistance, we investigated the role of post-translational modifications on epigenetic regulators, focusing on the phosphorylation-dependent control of histone demethylase KDM6A. Through integrated phosphoproteomics and functional genomics in HNSCC models, we identified phosphorylation of KDM6A at Ser829 as a master regulatory switch. Mechanistically, phosphorylation at Ser829 subverts KDM6A function, leading to a global accumulation of repressive H3K27me3 marks. This establishes an epigenetic program that locks key pyroptosis effector genes (*GSDMD*, *NLRP3*) in a transcriptionally refractory, heterochromatic state. To pharmacologically dismantle this barrier, we developed a novel lipid nanoparticle (LNP) system encapsulating mRNA encoding a phosphorylation-defective *KDM6A*^*S829A*^ variant. This strategy functionally reverses the oncogenic epigenetic state, restoring endogenous demethylase activity specifically at pyroptosis loci and reigniting the cell death pathway. In vivo, a single administration of *KDM6A*^*S829A*^ mRNA inhibited tumor growth by 39% (*p* < 0.01). Strikingly, when combined with anti-PD-1, this strategy achieved a synergistic 79% tumor reduction (*p* < 0.0001), characterized by robust CD8^+^ T cell infiltration. Our work identifies KDM6A phosphorylation as a druggable epigenetic-immune checkpoint and establishes mRNA-based modulation of enzyme post-translational status as a transformative paradigm to potentiate ICB in HNSCC.

**Figure:**
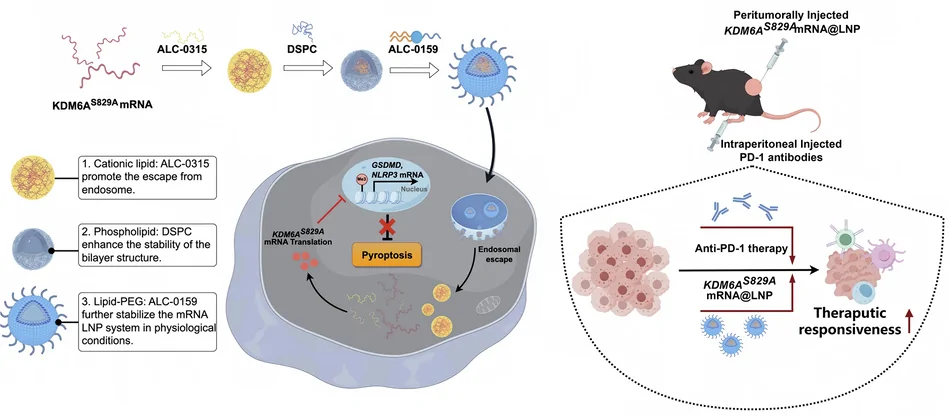

## Introduction

Immune checkpoint blockade (ICB) has revolutionized cancer therapy, significantly improving clinical outcomes in malignancies such as melanoma, non-small cell lung cancer (NSCLC), hepatocellular carcinoma (HCC), renal cell carcinoma (RCC), and head and neck squamous cell carcinoma (HNSCC) [1–5]. Despite these advances, challenges persist in the long-term efficacy of ICB, including the prevalence of immunologically “cold” tumors and acquired resistance [6]. Current strategies to enhance ICB, including combinations with radiotherapy, chemotherapy, tumor vaccines, or adoptive cell therapies, face limitations due to heterogeneous responses and unresolved resistance mechanisms [7]. Therefore, there is a critical need to elucidate tumor-intrinsic and microenvironmental adaptations driving resistance, which may unveil novel therapeutic targets to optimize ICB outcomes.

Pyroptosis, a lytic programmed cell death pathway mediated by gasdermin proteins, generates potent inflammatory signals that may convert immunologically quiescent tumors into T cell-infiltrated niches, thereby synergizing with ICB [8, 9]. While preclinical studies demonstrate that forced gasdermin D (GSDMD) expression via mRNA delivery can induce pyroptosis, translational applications are hampered by transient transgene expression ( < 72 h), off-target cytotoxicity in normal tissues, and inability to sustain tumor-specific immune activation [10]. Moreover, single-agent GSDMD overexpression inadequately addresses the epigenetic silencing of pyroptosis-related genes (e.g., *NLRP3*, *GSDMD*) commonly observed in ICB-resistant tumors. These limitations underscore the necessity for precision strategies that durably rewire tumor cell death pathways while circumventing microenvironmental immunosuppression. Emerging evidence implicates histone modification dynamics as critical regulators of pyroptosis [11, 12]. For instance, H3K27me3 demethylases like Lysine specific demethylase 6 A (KDM6A) modulate chromatin accessibility at inflammasome and gasdermin loci, positioning them as potential therapeutic nodes [13]. However, no epigenetic targets have been clinically validated to enhance pyroptosis in ICB-resistant cancers. Key knowledge gaps persist in how post-translational modifications of epigenetic enzymes (e.g., phosphorylation, acetylation) alter their substrate specificity in tumors, whether pharmacologically targeting these modifications can reverse pyroptosis suppression without global chromatin disruption. Addressing these questions is essential for unlocking novel combination therapies to reignite anti-tumor immunity.

In this study, we performed phosphoproteomics, chromatin immunoprecipitation sequencing and functional genomics of HNSCC models to uncover key targets to trigger pyroptosis of tumor cells. Ser829 phosphorylation of the histone demethylase KDM6A was identified as a master regulatory switch of pyroptosis. To therapeutically target this node, we developed lipid nanoparticles (LNPs) encapsulating phosphorylation-defective *KDM6A*^*S829A*^ mRNA to restore pyroptosis in HNSCC cells. These results propose mRNA-based targeting of this residue as a strategy to overcome immunotherapy resistance in HNSCC.

## Materials and methods

### Animals

The male BALB/c nude mice and C57BL/6 J mice (6 weeks old) used in this study were housed in the SPF-grade animal breeding facility at the Ninth People’s Hospital, affiliated with Shanghai Jiao Tong University School of Medicine. The animals were maintained at a stable room temperature (22–25 °C) with unrestricted access to food and water and were subjected to a 12-h light/dark cycle each day. Following quarantine clearance, all experimental animals were acclimated in the breeding facility for one week before the commencement of the experiments. All animal studies were conducted in strict accordance with the ARRIVE reporting *guidelines* and the relevant ethical regulations of the hospital [14]. The experimental protocols were reviewed and approved by the Animal Care and Use Committee of Shanghai Ninth People’s Hospital (SH9H-2025-A1561-1).

### Cell culture

HN6 (RRID: CVCL_5516), MOC1 (RRID: CVCL_ZD32) and SCC7 (RRID: CVCL_V412) cell lines were used for this study. HN6 cell line originated from male patients with oral squamous cell carcinoma. SCC7 and MOC1 cell lines originated from murine squamous cell carcinoma. HN6 cell line was kindly gifted by Professor Mao Li from the University of Maryland. The murine tumor cell line MOC1 was purchased from the Dana-Farber Cancer Institute. SCC7 cell line was obtained from the Cell Resource Center, Peking Union Medical College. All cell lines were confirmed mycoplasma-negative by PCR/qPCR testing and authenticated within the past 3 years using STR profiling. HN6 and SCC7 cell lines were cultured in high-glucose DMEM medium (BasalMedia, China) supplemented with 10% fetal bovine serum (FBS; Cellmax, China) and 1% penicillin–streptomycin (PS; TBD, China). While the MOC1 cell line was cultured in IMDM/F12 = 2:1 medium (BasalMedia, China) supplemented with 5 mg/L insulin, 40 μg/L hydrocortisone, 5 μg/mL EGF, 10% FBS, and 1% PS. Cells were incubated at 37 °C in an atmosphere of 5% CO_2_.

### Patient samples

The clinicopathological features of HNSCC patients from the Department of Oral Pathology of Shanghai Ninth People’s Hospital were collected and retrospectively analyzed, from which 100 paraffin-embedded primary specimens were collected for histological analyses. Tumor tissues from patients with HNSCC were collected between July 2017 and January 2019 along with complete follow-up data. The TNM staging criterion was based on the latest American Joint Committee on Cancer (AJCC) staging. Detailed information on KDM6A-pSer829 and total KDM6A expression analysis and the corresponding clinical characteristics of patients with HNSCC is provided in Table S1. The use of human specimens in this study was approved by the Institutional Research Ethics Committee of Shanghai Ninth People’s Hospital (SH9H-2024-T281-1), and a waiver of informed consent was granted.

### Phosphoproteomics

Establishment of the mouse subcutaneous xenograft model was described in a previous study [15]. Male C57BL/6 J mice aged 6 weeks were selected in this experiment. 100 μL of cell suspension (500,000 MOC1 cells re-suspended in PBS) was subcutaneously injected into the flank of mice after general anesthesia with 1% pentobarbital sodium. On the 4th day, after confirming tumor establishment, the mice were divided into two groups and underwent the indicated treatment. PD-1 monoclonal antibodies and IgG isotype monoclonal antibodies (10 mg/kg; Bio X Cell, USA) were intraperitoneally injected into the mice three times a week, respectively. Tumor sizes and body weight were measured three times a week. Once the tumor volume exceeded 1000 mm^3^, euthanasia by CO_2_ inhalation was performed. Subsequently, the phosphoproteomics were conducted by Applied Protein Technology, and GO enrichment analysis was performed by Metascape [16].

### Quantitative real-time PCR (qPCR)

The qPCR assay was conducted in accordance with the previous study [17]. Total RNA was extracted using the RNA-Quick Purification Kit (ESScience, China) following the manufacturer’s instructions, and cDNA was synthesized with the PrimerScript RT Reagent Kit (Takara, Japan). qPCR reactions were performed on an ABI StepOne real-time PCR system (Life Technologies, USA) using the Hieff UNICON® qPCR SYBR Green Master Mix (Yeasen, China). The amplified PCR products were quantified and normalized against β-Actin as the reference gene. Primer concentrations were set at 0.25 μM, and details of primer sequences and other consumables used in the qPCR assay are provided in the Supplementary Materials.

### Western blotting assay

Western blotting was used to measure the protein expression levels in cells as described in the previous study [18]. For the Western blotting assay, cells were lysed with RIPA lysis buffer (NCM Biotech, China) and subjected to protein quantification using the BCA Protein Assay Kit (Thermo Fisher Scientific, USA). The protein samples were added with a quarter volume of 5 × loading buffer (NCM, China) and incubated at 105 °C for 15 min. Then the protein samples were separated by polyacrylamide gels (Genshare Biological, China) and transferred to 0.22 μm polyvinylidene fluoride (PVDF) membranes (Merck Millipore, USA). The blots were blocked with 5% skimmed milk in TBST for 1 h at room temperature. After washing with TBST for 15 min, the blots were incubated with specific primary antibodies overnight at 4 °C. Afterward, the membranes were probed with HRP-conjugated secondary antibody (Beyotime, China; A0208, 1:200) and visualised with Immobilon Forte Western HRP Substrate (Merck Millipore, USA) and Amersham Imager 600 system (General Electric Company, USA). β-actin was used as a loading control. The primary antibodies used in this study are anti-KDM6A (CST, USA; 1:1000), anti-KDM6A-pSer829 (GeneScript, China; 1:1000), and anti-β-Actin (Abclonal, China; AC026, 1:200000).

### CCK-8 assay

Cells were seeded in triplicate into 96-well plates at a density of 1,000 cells per well. CCK-8 reagent (CellCook, China) was diluted 10-fold with FBS-free medium and added to replace the original medium (100 μL per well). After incubating at 37 °C for 2 h, the optical density (OD) of each well was measured at 450 nm using a microplate reader (SpectraMax i3, Molecular Devices, USA).

### Colony formation assay

MOC1 cells were seeded in triplicate into 6-well plates at a density of 100 cells per well for one week. When visible colonies appear on the plate, discard the supernatant and gently wash the cells with PBS. 4% paraformaldehyde was added to fix the colonies for 20 min. After washing with PBS, crystal violet staining solution was added and incubated for 20 min. Subsequently, the colonies were washed with slow-running water to remove the crystal violet staining solution. The plate was photographed after drying at 37 °C.

### Detection of LDH activity

The experiment was conducted using HN6, SCC7, and MOC1 cells. Cells were incubated with vector or mRNA@LNPs for 48 h. Subsequently, procedures were carried out according to the manufacturer’s instructions, with LDH release reagent and LDH detection working solution prepared freshly. The OD value was measured at 450 nm using a microplate reader (SpectraMax i3, Molecular Devices, USA).

### mRNA@LNPs generation

In vitro transcription (IVT) of mRNA was synthesized using T7 polymerase-conducted transcription. Plasmids carrying open reading frames (ORFs), 5 ‘and 3’ untranslated regions (UTRs, 3 ‘UTR with polyA tail) and T7 promoter. Linearized template DNA was digested by restriction enzymes BspQI, followed by purification. Linearized plasmid DNA was used as a template for an in vitro reaction catalyzed by T7 RNA polymerase. The reaction mixture contained ATP, CTP, GTP, N1-Me-Pseudo-UTP, a trinucleotide cap analog (CleanCap®), pyrophosphatase, and Mg2^+^. The IVT reaction was performed with pH 8.0 TRIS buffer at 37 °C for 3 h. Then the IVT transcripts were treated with DNase I and followed by purification with Magnetic beads. The final mRNA products were diluted in DNase/RNase-free distilled water and stored at –80 °C.

The mRNA@LNPs were prepared using microfluidic technology. In brief, a lipid mixture containing ionizable lipid, DSPC, cholesterol, and ALC-0159 was dissolved in ethanol at a specific molar ratio to form the organic phase. The LNPs encapsulation process involved mixing the organic phase with mRNA in an aqueous solution using microfluidic system (NanoAssemblr Ignite, Precision) at an N/P ratio of 6 and a flow rate of 1:3. The encapsulated sample was promptly diluted and subjected to buffer exchange in 10 mM Tris buffer (pH 7.4) to remove residual ethanol by multiple rounds of ultrafiltration/diafiltration using centrifuge tubes (MWCO 100 kDa, Cat# VS15T42, Sartorius). The final mRNA-LNPs sample was adjusted to 100 μg/mL in Tris buffer containing a cryoprotectant, filtered through a 0.22 μm PES filter (0.22 μm, Cat# SLGPR33RS, Merck), and stored at –80 °C for subsequent use.

### Immunofluorescence

For cell immunofluorescence assays, HN6, SCC7, and MOC1 cell lines treated with *GFP* mRNA@LNPs for 24 h were cultured in confocal dishes and then fixed with 4% paraformaldehyde. Nucleus was visualized by 4′,6-diamidino-2-phenylindole (DAPI; Beyotime, China; 1:100) staining. Images were obtained with a ZEISS LSM880 scanning confocal microscope (ZEISS, Germany).

### Establishment and treatment of mouse subcutaneous xenograft model

Male BALB/c nude mice and C57BL/6 mice aged 6 weeks were selected in this experiment. For BALB/c nude mice, 100 μL of cell suspension (500,000 HN6 cells re-suspended in PBS) was subcutaneously injected into the flank of nude mice after general anesthesia with 1% pentobarbital sodium. Tumor growth was monitored by measuring the tumor dimensions (length, width, and height) three times a week using calipers at consistent times of day. On the 5th day, after confirming tumor establishment, the nude mice were randomly divided into two groups by body weight and underwent the indicated treatment (*n* = 6 per group). *KDM6A*^*S829A*^ mRNA@LNPs (0.5 μg/g) were peritumorally injected into the tumor. Once the tumor volume exceeded 1500 mm^3^, euthanasia by CO_2_ inhalation was performed. Tumors were photographed, weighed, and then fixed with 4% paraformaldehyde for histological analysis.

For C57BL/6 mice, 100 μL of cell suspension (500,000 MOC1 cells re-suspended in PBS) was subcutaneously injected into the flank of mice after general anesthesia with 1% pentobarbital sodium. Tumor growth was monitored by measuring the tumor dimensions (length, width, and height) three times a week using calipers at consistent times of day. After confirming tumor establishment, the mice were randomly divided into different groups by body weight and underwent the indicated treatment. *KDM6A*^*S829A*^ mRNA@LNPs, *KDM6A*^*WT*^ mRNA@LNPs or control mRNA@LNPs (0.5 μg/g) were peritumorally injected into the tumor. PD-1 monoclonal antibodies and IgG isotype monoclonal antibodies (10 mg/kg; Bio X Cell, USA) were intraperitoneally injected into the mice, respectively. Once the tumor volume exceeded 1,500 mm^3^, euthanasia by CO_2_ inhalation was performed. Tumors were photographed, weighed, and then prepared for flow cytometry.

### H&E staining and immunohistochemistry (IHC)

For H&E staining, paraffin-embedded sections (3 μm thick) were deparaffinized and rehydrated. The nuclei were counterstained with hematoxylin, followed by dehydration, while eosin was used to stain the cytoplasm. The slides were then sequentially submerged in graded ethanol and xylene before being mounted with coverslips. For IHC, paraffin-embedded 3 μm thick sections were deparaffinized, rehydrated, and subjected to heat-induced antigen retrieval using a citric acid buffer. Endogenous peroxidase activity was blocked by immersion in 0.3% hydrogen peroxide. After blocking with 3% goat serum albumin, the sections were incubated with primary antibodies at 4 °C overnight. The following day, the sections were incubated with an HRP-conjugated secondary antibody (Beyotime, China; A0208, 1:400) at room temperature for 1 hour. Hematoxylin was used for nuclear counterstaining, followed by dehydration. The slides were then immersed in graded ethanol and xylene before being mounted with coverslips. Images were acquired using a panoramic slide scanner (Pannoramic MIDI/P250, 3DHISTECH, Hungary). The antibodies used in this experiment are anti-KDM6A (CST, USA; 33510, 1:200), anti-H3K27me3 (Abclonal, China; A2363, 1:200), anti-GSDMD (Abclonal, China; A20728, 1:200), anti-Caspase-1 (Selleck, China; F0461, 1:200).

### Flow cytometry

For apoptosis detection, MOC1 cells were transfected with the vector, KDM6A^S829A^ and KDM6A^829D^ for 48 h. After centrifugation at 1000 rpm for 3 min, the cells were resuspended in pre-cooled PBS. The cells were stained with an Annexin V-FITC apoptosis detection kit. Analysis was performed by flow cytometry (Beckman, CytoFLEXLX, USA) within 1 h. The data was processed using FlowJo software.

For intracellular uptake, HN6, SCC7 and MOC1 cells were seeded into 6-well plates and incubated with *GFP* mRNA@LNPs (1 μg/well) for 0, 12, 24 and 48 h, respectively. After centrifugation at 1000 rpm for 3 min, the cells were resuspended in pre-cooled PBS. Analysis was performed by flow cytometry (Beckman, CytoFLEXLX, USA) within 1 h. The data was processed using FlowJo software.

Isolation of tumor-infiltrating immune cells as described in our previous study. Briefly, the dissociated tumor tissues were analyzed by flow cytometry (Agilent, NovoCyte Penteon, USA). Live cells were incubated with Fc block (BioLegend, USA; 156603, 1:100) in 50 μL PBS with 1% FBS for 5 min on ice, followed by adding 0.2 μL LIVE/DEAD Dye (eBioscience, USA; 65-0865-14) for 15 min on ice. Then adding antibody-fluorochrome combinations: Anti-mouse CD11b BUV395, Anti-mouse F480 APC, Anti-mouse CD86 BV421, Anti-mouse CD206 PE, Anti-mouse CD11c PE-Cy7, Anti-mouse MHC-II BV786, Anti-mouse CD80 FITC, Anti-mouse CD45 BV510, Anti-mouse CD3 PE, Anti-mouse PD1 BV421, Anti-mouse CD8 PE-Cy7, Anti-mouse CD4 FITC, Anti-mouse CD25 APC, Anti-mouse Foxp3 PE, Anti-mouse CD45 FITC and Anti-mouse CD4 BV421. The flow cytometry gating strategy was provided in Figs. S7–S9. All the antibodies used in this study were listed in the Supplementary Materials.

### RNA-sequencing (RNA-seq) experiment and analysis

RNA-seq assay was performed by OEBiotech China. Total RNA was extracted from HN6 cells overexpressing different KDM6A mutants, and a cDNA library was prepared according to the standard Illumina RNA-seq instructions. Hisat2 was selected as the mapping tool because Hisat2 can generate a database of splice junctions based on the gene model annotation file. FeatureCounts v1.5.0-p3 was used to count the read numbers mapped to each gene. A cDNA library was prepared according to the standard Illumina RNA-seq instructions. Hisat2 was selected as the mapping tool because Hisat2 can generate a database of splice junctions based on the gene model annotation file. FeatureCounts v1.5.0-p3 was used to count the read numbers mapped to each gene.

### Chromatin immunoprecipitation-sequencing (ChIP-seq) experiment and analysis

CHIP-seq was performed as previously reported method [19]. HN6 overexpressed different mutants of KDM6A for 48 h. The EZ ChIP kit (CST, USA) was used for ChIP detection. Briefly, HN6 (1 × 10^7^ cells) were fixed with 1% formaldehyde and then neutralized with 0.125 M glycine. Cells were collected and cleaved in a cell lysis buffer containing a mixture of SDS and protease inhibitors. Soluble chromatin with an average length of 500 bp was obtained by ultrasonic treatment of the lysate. After 1:10 dilution in the dilution buffer, the chromatin solutions were pre-cleared and incubated with anti-H3K27me3 antibodies. Next, the mixtures were incubated overnight on a rotating platform at 4 °C. The immunocomplexes were captured by A protein A/G-Sepharose beads. After a large amount of washing, the bound DNA fragments were eluted. The libraries with different indexes were multiplexed and loaded on an Illumina HiSeq instrument according to manufacturer’s instructions (Illumina, San Diego, USA). Sequencing was carried out using a 2 × 150 paired-end (PE) configuration; image analysis and base calling were conducted by the HiSeq Control Software (HCS) + OLB + GAPipeline-1.6 (Illumina) on the HiSeq instrument. The sequences were processed and analyzed by GENEWIZ.

### Bioinformatic analysis

Transcriptome data of TCGA HNSCC were downloaded from the Genomic Data Commons (GDC) portal via TCGA biolinks following a standard protocol [20]. The RNA expression and survival analysis were performed in GEPIA2 [21]. The protein expression analysis was performed in LinkedOmicsKB [22]. Clinical data of anti-PD-1 treated patients was performed in KM Plotter [23]. The GO enrichment analysis was performed in Metascape [16]. Protein–protein interaction (PPI) network was performed in STING [24]. The correlation between *KDM6A* levels and immune cell infiltration was performed in TIMER [25]. The public dataset could be obtained as follows. Single-cell RNA-seq datasets: BCC & SCC (GSE123813), HNSCC (GSM7324295, GSE232240), melanoma (GSE120575), CCRCC (SCP1288). Spatial transcriptome datasets: HNSCC (GSE220978), cSCC (GSE144240), ccRCC (GSE175540), HGSOC (GSE211956), NSCLC (E-MTAB-13530), CRC (GSE225857). Samples were analyzed with the Seurat package (v4.4.0) in R-4.3.2. GSVA package (v1.50.5) was used for pyroptosis-related genes scoring and Spearman correlation coefficient was calculated.

### Statistical analysis

All data were presented as means ± standard error of mean (SEM). The normal distribution and homogeneity of the data were tested by SPSS. Unless otherwise stated, an unpaired or paired 2-tailed student’s *t* test was used to test the significance of differences between the two groups. Two-way analysis of variance (ANOVA) was used to compare the effects of different factors on mouse tumor volume. Survival analysis was plotted using the Kaplan-Meier method. Statistical analyses were conducted using GraphPad Prism Software. The exact sample size (n) for each experimental group/condition is given as a discrete number and unit of measurement. Our experimental sample size was selected according to preliminary studies in our laboratories or in similarly published research. For animal studies, a minimum of three mice per group was used, and the exact number of mice in each experiment is indicated in the corresponding Fig. legends. No data were excluded in the study. All in vivo experiments were randomized to body weight, and all replication attempts were successful. Blinding was performed for the IHC scoring analysis to prevent subjective bias in data collection. For data collection in other experiments, blinding was not applied because the investigator needs to know each treatment group to perform the experiments. *p* values less than 0.05 were considered statistically significant, the level of significance was set at *p* < 0.05 (*), *p* < 0.01 (**), *p* < 0.01 (***) or *p* < 0.001 (****).

## Results

### PD-1 blockade impairs KDM6A phosphorylation at Ser829 in HNSCC

**Fig. 1:**
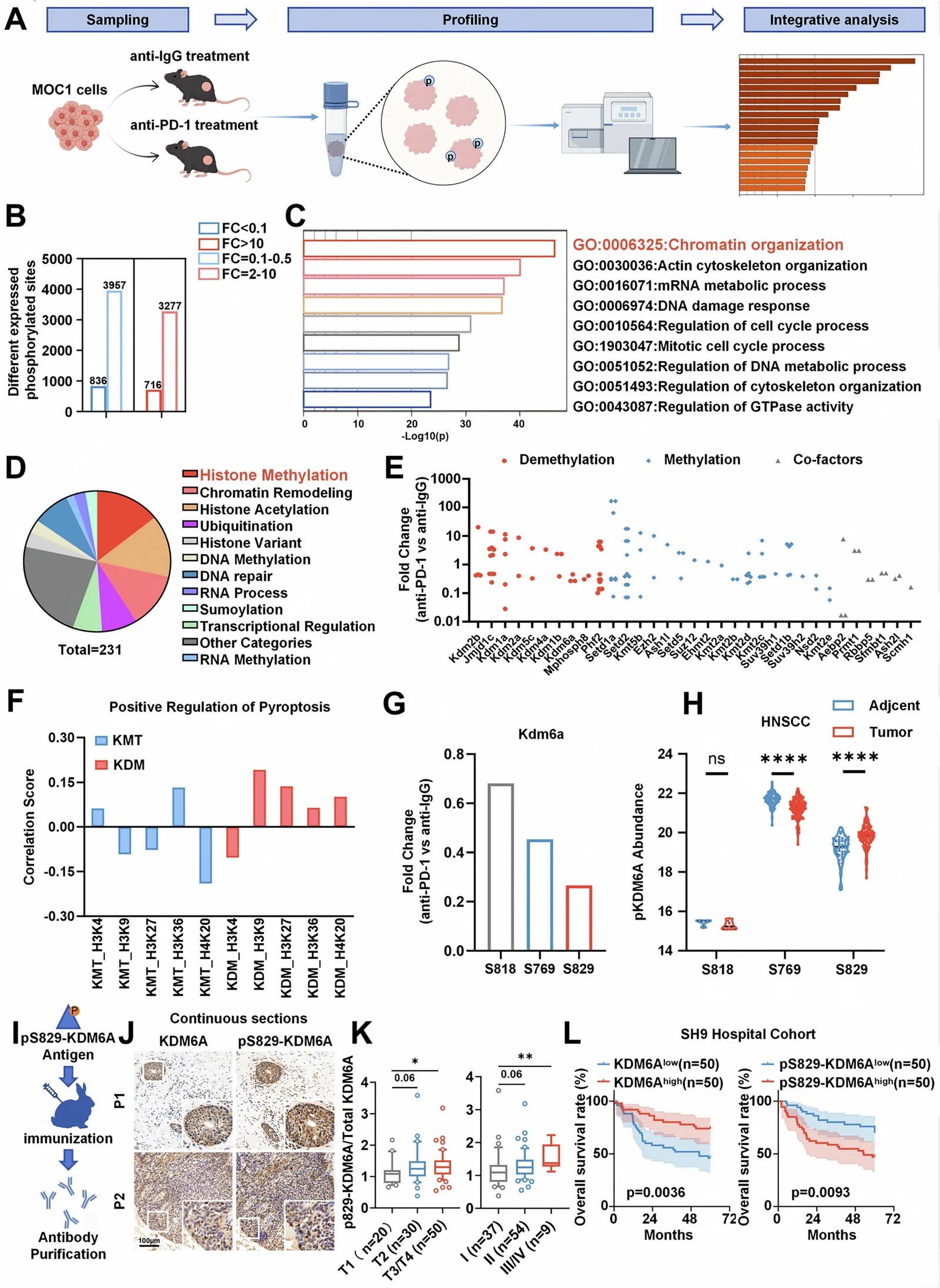
PD-1 blockade impairs KDM6A phosphorylation at Ser829 in HNSCC. **A** Flow diagram illustrated phosphoproteomics strategies for identifying potential phosphorylated proteins in HNSCC treated with anti-PD-1 therapy. **B** Differential expression of phosphorylated sites in HNSCC treated with anti-PD-1 therapy. **C** Enrichment analysis of the phosphorylated proteins in HNSCC tissues treated with anti-PD-1 therapy (FoldChange = 2). **D** Proportion of sub-pathways in “chromatin organization” enriched by GO analysis. **E** The expression levels of histone methylation-related proteins in HNSCC treated with anti-PD-1 therapy. **F** Correlation analysis between the expression levels of *KDM6A* and pyroptosis-related genes in HNSCC from TCGA database. **G** The expression levels of phosphorylated sites of KDM6A in HNSCC treated with anti-PD-1 therapy. **H** The expression levels of phosphorylated sites of KDM6A in HNSCC from LinkedOmicsKB; Ns, no significance; ****p < 0.0001. **I** Diagram illustrated the purification of anti- KDM6A-pSer829. **J** Immunohistochemistry analysis showed the expression of KDM6A and KDM6A-pSer829 in HNSCC patients, respectively. **K** Correlation of pS829-KDM6A (normalized to total KDM6A) abundance and clinical characteristics from HNSCC patients. **L** Kaplan-Meier survival analysis of OS in SH9 hospital cohort with HNSCC. Ns, no significance; *p < 0.1, **p < 0.01, ****p < 0.0001.

Phosphorylation plays an essential role in pyroptosis in cancer [26]. Therefore, to determine whether there are phosphorylated modifications related with pyroptosis in PD-1-blockaded tumors, we systematically mapped the phosphorylated proteins in MOC1 xenografts treated with PD-1 monoclonal antibodies or IgG isotype monoclonal antibodies by phosphoproteomic profiling (Fig. 1A). Compared with placebo group, we identified 3993 upregulated phosphorylation sites and 4,793 downregulated sites following PD-1 blockade (Fig. 1B). According to the GO enrichment analysis, the “Chromatin organization pathway” was significantly upregulated (*p* <0.01) (Fig. 1C), whereas “Histone Methylation” is the primary sub-pathway of “Chromatin organization pathway” (Fig. 1D). This pathway included 10 lysine demethylases (KDMs), 17 lysine transmethylases (KMTs) and 6 co-factors (Fig. 1E).

To identify whether site-specific histone methylation-related proteins have a correlation with pyroptosis in HNSCC, we performed comprehensive correlation analysis by calculating methylation sites and pyroptosis scores from the TCGA database. KMTs for H3K9 and H3K27 were negatively correlated with pyroptosis, while KDMs for H3K9 and H3K27 were positively correlated (Fig. 1F), indicating methylation of H3K9 and H3K27 was the potential switch for pyroptosis in tumor cells during PD-1 blockade. Previous studies have reported that epigenetic mechanisms, including H3K27me3-mediated transcriptional regulation, can modulate the expression and activation of pyroptosis-related molecules such as GSDMD and GSDME [11, 27–30]. Further bioinformatic analysis with protein-protein interactions (PPI) network uncovered that KDM6A, a histone H3K27 demethylase, was the most central target gene in PD-1 blockaded tumors [24] (Fig. S1). Moreover, *KDM6A* expression level positively correlated with pyroptosis-related genes in TCGA patients with HNSCC (Fig. S2), supporting KDM6A as a strong candidate to regulate pyroptosis. Therefore, we first examined the role of KDM6A in pyroptosis regulation and then further investigated whether phosphorylation of KDM6A affects this function in HNSCC.

KDM6A is known to erase H3K27me3 via its demethylase activity. To identify functional phosphorylation of KDM6A during PD-1 blockade, we further analyzed the phosphorylation of KDM6A. Our phosphoproteomic profiling detected three KDM6A phosphorylated sites (Ser769, Ser818, and Ser829), all of which showed reduced expression levels in PD-1 blockage-treated tumors compare to placebo control (Fig. 1G). We further identified KDM6A-pSer829 emerged as the only increased modification in tumor tissues compared to adjacent tissues from LinkedOmicsKB patients with HNSCC (Fig. 1H). Next, we generated and purified polyclonal antibodies targeting KDM6A-pSer829 by immunizing New Zealand rabbits for immunohistochemistry analysis (Fig. 1I). KDM6A-pSer829 band was significantly increased in HN6 cells expressing wild-type KDM6A rather than phosphor-deficient KDM6A^S829A^, supporting the specificity of this antibody for detecting pSer829 KDM6A (Fig. S3). In HNSCC tissues, both KDM6A and KDM6A-pSer829 were expressed in cancer cells (Fig. 1J). We further analyzed the relationship between clinical information and KDM6A-pSer829 abundance based on tissues of HNSCC and found that elevated levels of KDM6A-pSer829 (normalized to total KDM6A) increased the risk of tumor size and TNM stage (Fig. 1K), but not metastasis (Fig. S4). While higher KDM6A levels were correlated with better OS, higher KDM6A-pSer829 levels, which normalized by KDM6A levels, were correlated with worse OS in patients with HNSCC in the SH9 hospital cohort (Fig. 1L). These results indicate that KDM6A-pSer829 correlates with worse patient outcomes and may a potential regulator linking pyroptosis to histone methylation in response to PD-1 blockade.

### Dephosphorylation at Ser829 is essential for KDM6A to excite pyroptosis in HNSCC

**Fig. 2:**
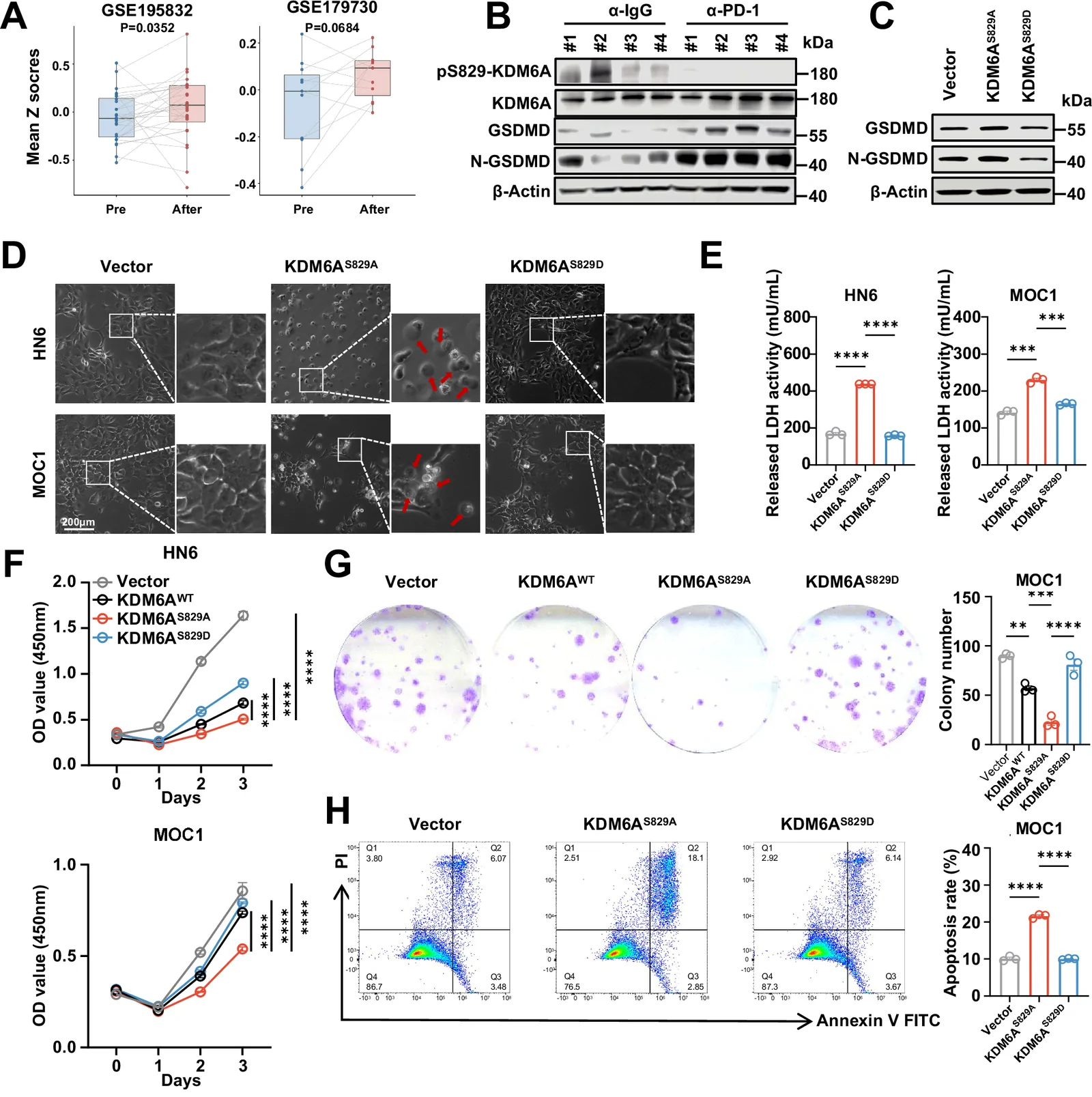
Dephosphorylation of KDM6A at pSer829 excites pyroptosis in HNSCC cells. **A** Increased expression of pyroptosis-related genes in HNSCC tissues after anti-PD-1 therapy. **B** Immunoblotting against pKDM6A, KDM6A, GSDMD and β-Actin in whole-cell lysates from MOC1 tumor tissues treated with PD-1 antibody (10 mg/kg; *n* = 4 per group). **C** Immunoblotting against GSDMD and β-Actin in whole-cell lysates from HN6 cells transient transfected with Vector, KDM6A^S829A^ or KDM6A^S829D^ for 48 h. **D** Representative images of HN6 and MOC1 cells transient transfected with Vector, KDM6A^S829A^ or KDM6A^S829D^ for 48 h. **E** Released LDH activity of HN6 and MOC1 cells transient transfected with Vector, KDM6A^S829A^ or KDM6A^S829D^ for 48 h (*n* = 3 per group). **F** CCK-8 assay detected the proliferation of HN6 and MOC1 cells transient transfected with Vector, KDM6A^WT^, KDM6A^S829A^ or KDM6A^S829D^ for 1-3 days (n = 5 per group). **G** Colony formation assay and quantification of colony numbers detected the proliferation of MOC1 cells transient transfected with Vector, KDM6A^WT^, KDM6A^S829A^ or KDM6A^S829D^ for 48 h (*n* = 3 per group). **H** Apoptosis detected by flow cytometry analysis of MOC1 cells transiently transfected with Vector, KDM6A^S829A^ or KDM6A^S829D^ for 48 h (*n* = 3 per group). **p < 0.01, ***p < 0.001, ****p < 0.0001.

To further confirm whether PD-1 blockade tigger pyroptosis in HNSCC, we analyzed the mean Z-scores of pyroptosis-related genes (Supplementary file 1) expression in HNSCC tissues treated with PD-1 blockade. Two independent bulk RNA-seq data of HSNCC cohorts with paired pre/after PD-1 blockade (GSE179730 and GSE195832) were included in this analysis, and we found a higher pyroptosis-related genes expression in tumor tissues after PD-1 blockade (Fig. 2A). Moreover, we established MOC1 subcutaneous xenografts model treated with anti-PD-1 therapy. The tumor tissues were collected and performed western blotting assay. The results showed that cleaved N-terminal GSDMD was significantly up-regulated in the tumor tissues treated with anti-PD-1 therapy (Fig. 2B). Together, these results demonstrate that PD-1 blockade triggers pyroptosis in HNSCC tissues.

Although previous studies have demonstrated its role in suppressing cell proliferation, it is still unknown whether KDM6A can induce pyroptosis in HNSCC [31]. To evaluate whether KDM6A phosphorylation at Ser829 changes cell pyroptosis, we overexpressed phosphor-mimic KDM6A^S829D^ and phosphor-deficient KDM6A^S829A^ in HNSCC cell lines, including human HN6 cells and murine MOC1 cells. Western blotting assay showed increased NLRP3 and cleaved N-terminal GSDMD in cells overexpressing KDM6A^S829A^ rather than KDM6A^S829D^ (Fig. 2C). Microscopic examination also showed the characteristic pyroptotic bubble-like morphology in both HN6 and MOC1 cells expressing KDM6A^S829A^ rather than KDM6A^S829D^ (Fig. 2D). Meanwhile, higher lactate dehydrogenase (LDH) levels were detected in the culture medium of KDM6A^S829A^-expressing cells compared to the KDM6A^S829D^ group (Fig. 2E). We further examined the proliferation of cells overexpressing phosphor-mimic KDM6A^S829D^ or phosphor-deficient KDM6A^S829A^. Consistent with its tumor-suppressive function, cells expressing KDM6A^S829A^ presented significantly decreased cell proliferation (Fig. 2F) and colony formation (Fig. 2G) compared with cells expressing KDM6A^S829D^. Flow cytometry analysis revealed a marked increase in Annexin V and propidium iodide (PI) double-positive cells in MOC1 cells expressing KDM6A^S829A^, indicating enhanced cell death (Fig. 2H). These data support that dephosphorylation of Ser829 is essential for KDM6A to induce pyroptosis in HNSCC cells.

### KDM6A phosphorylation at Ser829 silence *NLRP3* and *GSDMD* expression via H3K27me3

**Fig. 3:**
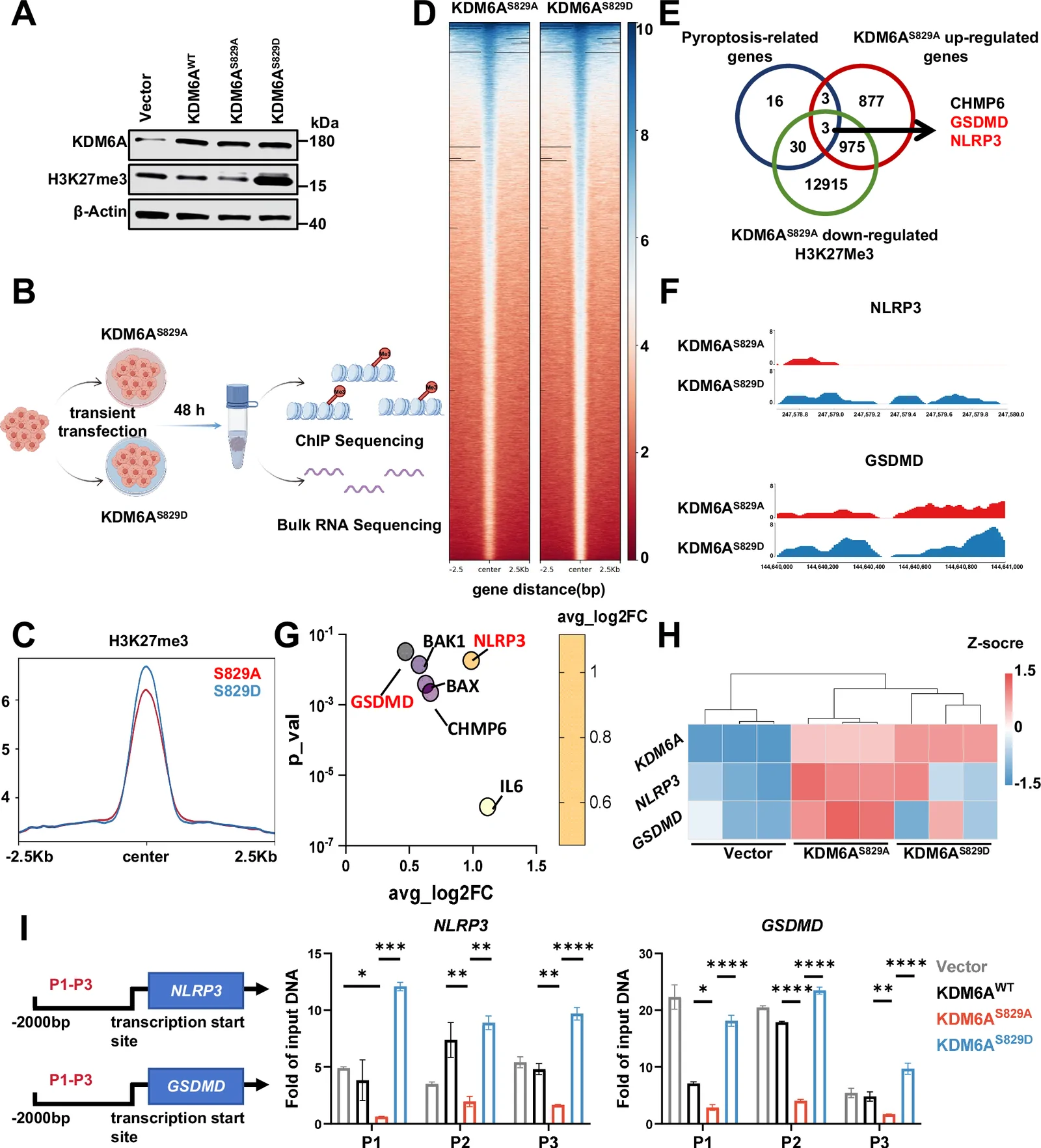
KDM6A phosphorylation at Ser829 silence *NLRP3* and *GSDMD* expression via H3K27me3. **A** Immunoblotting against KDM6A, H3K27me3 and β-Actin in whole cell lysates from HN6 cells transient transfected with Vector, KDM6A^WT^, KDM6A^S829A^ or KDM6A^S829D^ for 48 h. **B** Diagram illustrates the sample processing for RNA-seq and CHIP-seq. **C** Normalized CHIP-seq signals for H3K27me3 in HN6 cells transient transfected with KDM6A^S829A^ or KDM6A^S829D^ for 48 h. **D** Heatmap of the signals for H3K27me3 in HN6 cells transient transfected with KDM6A^S829A^ or KDM6A^S829D^ for 48 h. **E** The pyroptosis-related genes regulated by KDM6A^S829A^ displayed by CHIP-seq and RNA-seq. **F** Normalized CHIP-seq signals for H3K27me3 at the *GSDMD* and *NLRP3* locus in HN6 cells transient transfected with KDM6A^S829A^ or KDM6A^S829D^ for 48 h. **G** The expression of *GSDMD* and *NLRP3* mRNA were significantly up-regulated in HN6 cells transient transfected with KDM6A^S829A^ for 48 h. **H** The expression of *KDM6A*, *NLRP3* and *GSDMD* mRNA detected by qPCR in MOC1 cells transient transfected with Vector, KDM6A^S829A^ or KDM6A^S829D^ for 48 h (*n* = 3 per group). **I** CHIP-PCR detected the enrichment of H3K27me3 in the promoter region of *NLRP3* and *GSDMD* in HN6 cells transient transfected with Vector, KDM6A^S829A^ or KDM6A^S829D^ for 48 h (*n* = 3 per group). Ns, no significance; *p < 0.1, **p < 0.01, ***p < 0.001, ****p < 0.0001.

As KDM6A is a histone H3K27me3 demethylase, we next examined whether phosphorylation at S829 affects its ability to regulate H3K27me3 levels. As expected, overexpression of KDM6A^WT^ and KDM6A^S829A^ reduced H3K27me3 levels compared with the control group. In contrast, the KDM6A^S829D^ mutant showed an impaired ability to reduce H3K27me3, suggesting that S829 phosphorylation impaired the demethylase-associated function of KDM6A (Fig. 3A). Subsequently, we performed RNA-seq and CHIP-seq in HN6 cells expressing KDM6A^S829A^ and KDM6A^S829D^ to identify the target genes that induce pyroptosis (Fig. 3B). Compared with cells expressing KDM6A^S829D^, the average read count frequency at the transcript start site (TSS) was significantly higher in cells expressing KDM6A^S829D^ (Fig. 3B, C). Moreover, we integrated RNA-seq and ChIP-seq approaches to identify critical genes accounting for pyroptosis in cells expressing KDM6A^S829A^ (Fig. 3D). Among overlapping differentially expressed genes, *GSDMD* and *NLRP3* emerged as the upregulated mRNAs with lower H3K27me3 levels at promoters in cells expressing KDM6A^S829A^ (Fig. 3E, F). Meanwhile, qPCR assay confirmed that up-regulated expression of *GSDMD* and *NLRP3* mRNA in MOC1 cells expressing KDM6A^S829A^ compared to KDM6A^S829D^ (Fig. 3H). CHIP-PCR assay also indicated that lower H3K27me3 levels at the promoter region of *GSDMD* and *NLRP3* in HN6 cells expressing KDM6A^S829A^ compared to KDM6A^S829D^ (Fig. 3I). Taken together, these results confirm that phosphorylation of KDM6A at Ser829 increased H3K27me3 levels, leading to higher H3K27me3 deposition and lower *GSDMD* and *NLRP3* mRNA expression.

### *KDM6A* expression positively correlates with responsiveness to PD-1 blockade of cancer patients

**Fig. 4:**
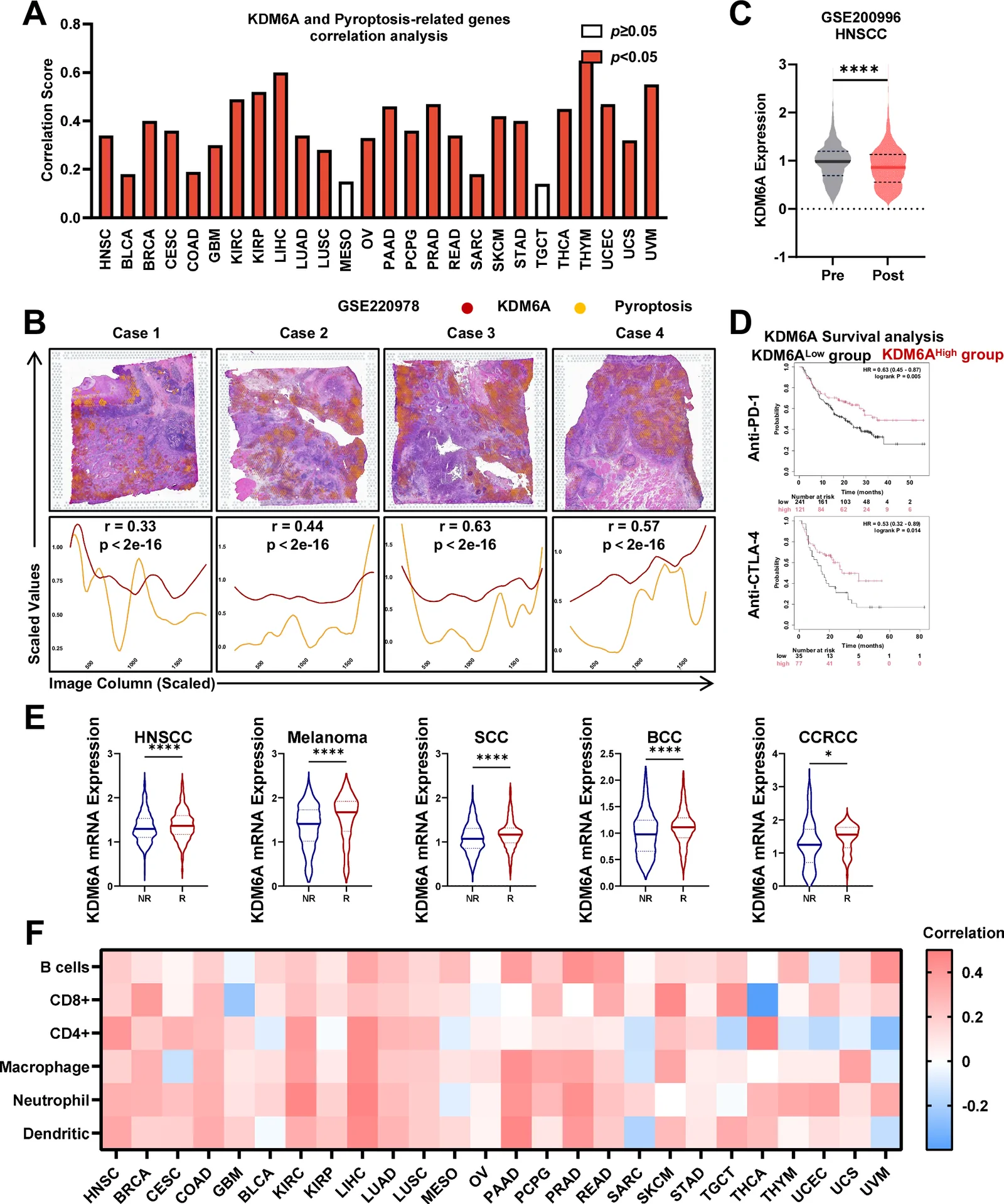
*KDM6A* expression positively correlates with responsiveness to PD-1 blockade of HNSCC patients. **A** Correlation analysis between *KDM6A* and pyroptosis-related genes in various cancers from TCGA database. **B** Colocalization of the spatial distribution of *KDM6A* mRNA and pyroptosis-related genes in HNSCC. **C** The expression levels of *KDM6A* were downregulated in HNSCC tissues after anti-PD-1 therapy. **D** Survival analysis for KDM6A in pan-cancers treated with anti-PD-1 or anti-CTLA-4 therapies. **E** The expression levels of KDM6A in different sensitivity of anti-PD-1 therapy. **F** Correlation analysis between KDM6A and infiltrated immune cells in various cancers. *p < 0.1, ****p < 0.0001.

Although the above results demonstrated that KDM6A^S829A^ has a robust anti-tumor effect by triggering pyroptosis of HNSCC cells, it is still unknown whether *KDM6A* mRNA levels correlate with pyroptosis in different cancer types. Therefore, to validate whether *KDM6A* expression positively correlates with pyroptosis-related genes (Supplementary file 1) in other cancer types, we calculated the correlation score on 26 different cancer types from the TCGA database. Interestingly, almost all types of cancers showed positive correlation between *KDM6A* mRNA levels with pyroptosis-related genes (Fig. 4A). We further conducted the spatial transcriptome analysis to determine whether the expression between *KDM6A* and pyroptosis-related genes were colocalization. The ssGSEA method, which employs a non-parametric approach to independently calculate enrichment scores for a specified gene set within each sample, was performed to calculate the pyroptosis score for each spot and jointly visualized with *KDM6A* expression levels [17, 32]. Red and orange points denote regions of high pyroptosis score and high *KDM6A* expression, respectively. For each spatial spot, the molecular neighborhood was defined as the six spatially adjacent spots surrounding it, forming a local hexagonal lattice structure that represents the immediate molecular microenvironment. The Spearman correlation coefficient (r) quantified the association between *KDM6A* expression levels within each molecular neighborhood and the corresponding pyroptosis score, with the *p*-value representing the statistical significance of this correlation. Remarkably, *KDM6A* expression and the pyroptosis score exhibited a moderate to strong positive correlation in more than half of the samples, suggesting that *KDM6A* may be functionally linked to pyroptosis in HNSCC tissues (Fig. 4B). Furthermore, we evaluated other 5 types of cancer (cSCC, ccRCC, HGSOC, NSCLC and CRC), and the results also indicated that KDM6A mRNA and pyroptosis-related genes were significantly co-localized in tumor tissues (Fig. S5). Moreover, the expression of *KDM6A* was positively associated with infiltration of immune cells in almost all type of cancers from TIMER [25] (Fig. 4F). These results suggested that KDM6A induced pyroptosis and promoted immune cells infiltration in examined different cancer types.

Next, we assessed whether *KDM6A* can be used as a potential predictive factor for the responsiveness of PD-1 blockade. Survival analysis showed that the high expression levels of *KDM6A* were correlated with better outcomes after treating with anti-PD-1 or anti-CTLA-4 therapies from KMplotter [23] (Fig. 4D). High expression of *KDM6A* patients showed improved overall survival compared to the low expression group in various types of cancer (Fig. S6). Interestingly, compared with pre-treatment, *KDM6A* expression was downregulated in the HNSCC tissues after anti-PD-1 therapy (Fig. 4C). Meanwhile, compared with the no-response (NR) group, the higher expression of *KDM6A* was detected in the response (R) group (Fig. 4E). In summary, the expression level of *KDM6A* is a potential factor to predict the responsiveness of PD-1 blockade, and higher *KDM6A* expression of tumor may have a better responsiveness to anti-PD-1 therapy.

### Delivery of phosphor-deficient *KDM6A*^*S829A*^ mRNA via LNPs in vitro

**Fig. 5:**
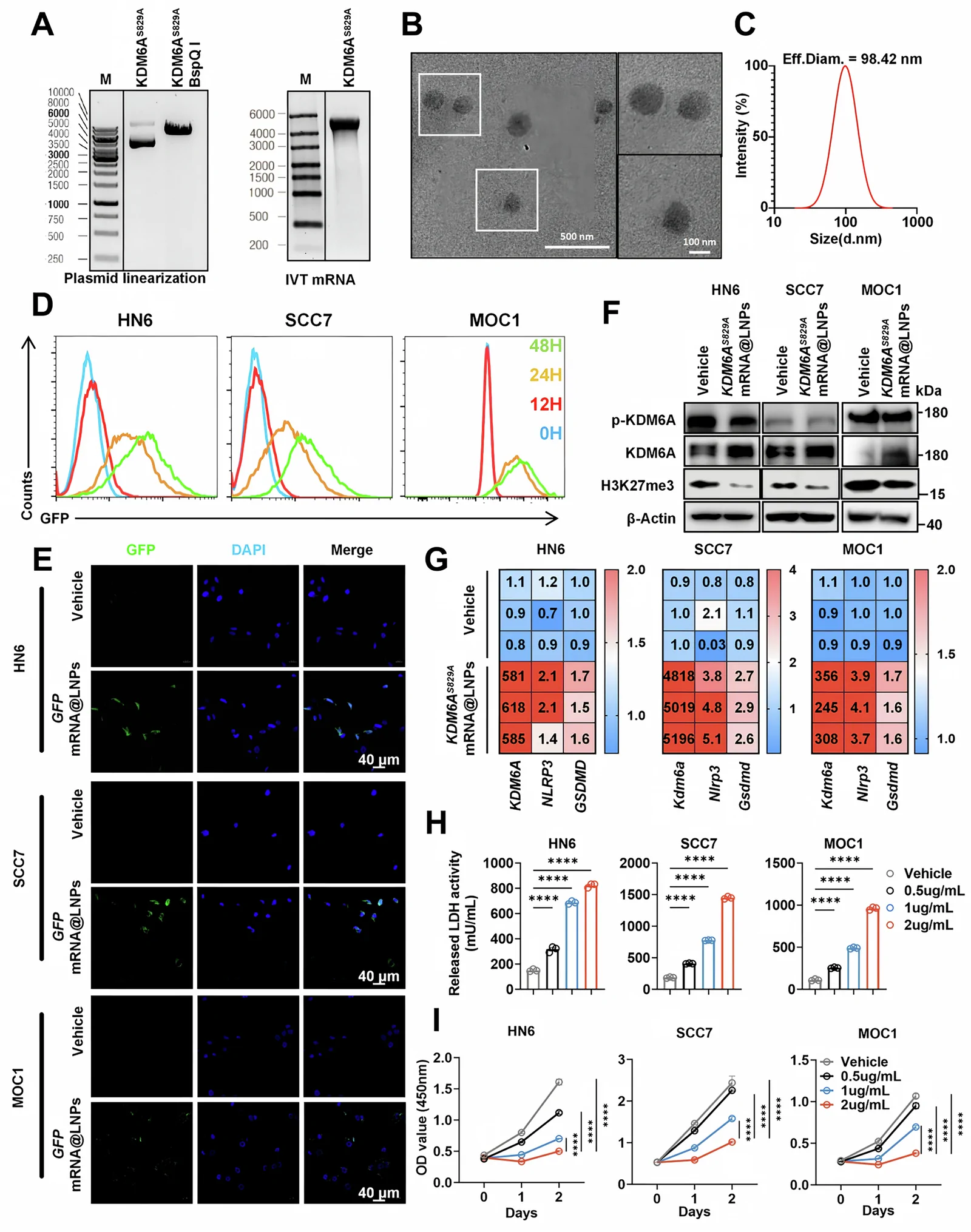
Lipid nanoparticles (LNPs) for *KDM6A*^*S829A*^ mRNA delivery to HNSCC in vitro. **A** After treatment with BspQ I, linearized plasmids were purified by phenol-chloroform before in vitro transcription (IVT). **B** Transmission electron microscopy and the average diameter for phosphor-deficient *KDM6A*^*S829A*^ mRNA@LNPs. **C** Dynamic light scattering analysis identified the average diameter for *KDM6A*^*S829A*^ mRNA@LNPs. **D** Fluorescence intensity detected by flow cytometry in HN6, SCC7 and MOC1 cells treated with *GFP* mRNA@LNPs (1 μg/mL). **E** Representative images of HN6, SCC7 and MOC1 cells treated with *GFP* mRNA@LNPs (1 μg/mL) for 48 h. **F** Immunoblotting against p-Kdm6a, Kdm6a, H3K27me3 and β-Actin in whole-cell lysates from HN6, SCC7 and MOC1 cells treated with vehicle or *KDM6A*^*S829A*^ mRNA@LNPs (1 μg/mL) for 48 h. **G** Heatmap of the expression of *KDM6A*, *NLRP3* and *GSDMD* mRNA detected by qPCR after incubating with *KDM6A*^*S829A*^ mRNA@LNPs (1 μg/mL) for 48 h (*n* = 3 per group). **H** CCK-8 assay detected the proliferation of HN6, SCC7 and MOC1 cells treated with *KDM6A*^*S829A*^ mRNA@LNPs in differential concentrations (0.5, 1 and 2 μg/mL) for 48 h (*n* = 3 per group). **I** Released LDH activity of HN6, SCC7 and MOC1cells treated with *KDM6A*^*S829A*^ mRNA@LNPs in differential concentrations (0.5, 1 and 2 μg/mL) for 1-2 days (*n* = 4 per group). Ns, no significance; *p < 0.1, ***p < 0.001, ****p < 0.0001.

LNPs can deliver exogenous mRNA for translation, thus largely avoiding host genome integration and eliciting faster and more predictable protein expression [33]. Therefore, it is necessary to examine whether the delivery of *KDM6A*^*S829A*^ mRNA can be used as a universal strategy to trigger pyroptosis in HNSCC. We generated *KDM6A*^*S829A*^ mRNA with in vitro transcript method (Fig. 5A) and packaged them into LNPs by the nanoprecipitation method (Fig. 5B). The average diameter of *KDM6A*^*S829A*^ mRNA@LNPs was 98.42 nm and maintained in phosphate-buffered saline (PBS) (containing 10% serum) at 37 °C, indicating acceptable stability of these engineered LNPs (Fig. 5C). We next evaluated the efficacy of HNSCC cells to uptake engineered mRNA@LNPs. The fluorescence intensity significantly increased after cells were incubated with *GFP* mRNA@LNPs for 24 h, indicating mRNA has been delivered into HNSCC cells and translated to proteins (Figs. 5D, E). Meanwhile, immunoblotting assays showed that the delivery of *KDM6A*^*S829A*^ mRNA@LNPs increased the total KDM6A protein with lower H3K27me3 levels compared to the vehicle group (Fig. 5F). These data demonstrated that *KDM6A*^*S829A*^ mRNA has been delivered into HNSCC cells and translated into protein.

Subsequently, to examine whether *KDM6A*^*S829A*^ mRNA@LNPs trigger pyroptosis, we incubated HN6, SCC7 and MOC1 cells with *KDM6A*^*S829A*^ mRNA@LNPs. Compared with the vehicle group, qPCR assay showed that the expression of *NLRP3* and *GSDMD* mRNA were significantly upregulated in the cells transfected with *KDM6A*^*S829A*^ mRNA@LNPs (Fig. 5G). We also examined the dosage effect of *KDM6A*^*S829A*^ mRNA@LNPs to inhibit cell proliferation. Concentration-dependent patterns were observed in the released LDH activity in the culture medium and inhibited cell proliferation of cells transfected with different dosages of *KDM6A*^*S829A*^ mRNA@LNPs (Fig. 5H, I). These data demonstrated that delivery of *KDM6A*^*S829A*^ mRNA@LNPs increases functional KDM6A to induce pyroptosis in HNSCC.

### Delivery of phosphor-deficient *KDM6A*^*S829A*^ mRNA via LNPs to trigger pyroptosis of HNSCC cells in vivo

**Fig. 6:**
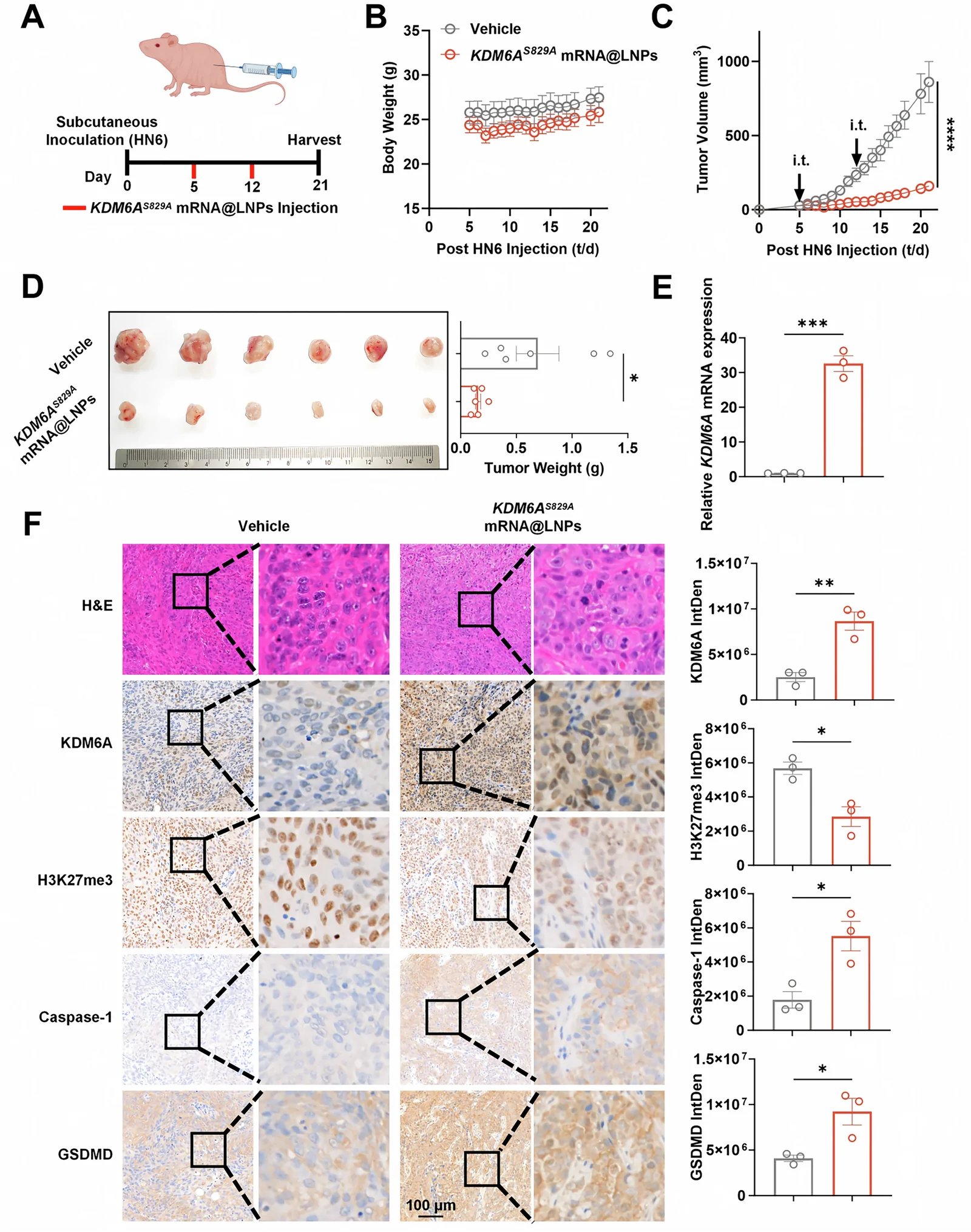
Delivering *KDM6A*^*S829A*^ mRNA@LNPs inhibits tumor growth and triggers pyroptosis of HNSCC in vivo. **A** Schematic diagram of establishment of subcutaneous xenografts model and *KDM6A*^*S829A*^ mRNA@LNPs injection (0.5 μg/g). **B** Quantification of body weight at indicated days after the mice were implanted with HN6 cells (*n* = 6 per group). **C** Quantification of tumor volume at indicated days after the mice were implanted with HN6 cells (*n* = 6 per group). **D** Images of tumors in the HN6 subcutaneous model with different treatments, and the quantification of tumor weight at the end of treatment. **E** The expression of *KDM6A* mRNA detected by qPCR in tumor tissues treated with vehicle or *KDM6A*^*S829A*^ mRNA@LNPs (*n* = 3 per group). **F** H&E staining and immunohistochemistry analysis showed the expression levels of KDM6A, H3K27me3, Caspase-1 and GSDMD in tumors treated with vehicle or *KDM6A*^*S829A*^ mRNA@LNPs and quantification of the expression respectively (*n* = 3 per group). *p < 0.1, **p < 0.01, ***p < 0.001.

Inspired by the anti-proliferative effects of *KDM6A*^*S829A*^ mRNA@LNPs against HNSCC cells in vitro, we examined the in vivo anti-tumor effects of *KDM6A*^*S829A*^ mRNA@LNPs upon injection into HN6 xenografts. After subcutaneous injection of HN6 cells for 5 days, mice with comparable tumor volumes were randomly divided into two groups and treated with PBS or *KDM6A*^*S829A*^ mRNA@LNPs via intratumoral administration (Fig. 6A). There was no significant change in body weight between the two groups (Fig. 6B), suggesting the biosafety of *KDM6A*^*S829A*^ mRNA@LNPs in vivo. Compared with the vehicle group, the intratumoral treatment with *KDM6A*^*S829A*^ mRNA@LNPs significantly inhibited the growth of tumor (Fig. 6C, D). To detect whether the abundance of KDM6A was increased by *KDM6A*^*S829A*^ mRNA@LNPs, we collected the xenograft tissues and performed qPCR and IHC examinations, respectively. Compared with vehicle group, the abundance of KDM6A signals were significantly increased both in mRNA (Fig. 6E) and protein (Fig. 6F) in *KDM6A*^*S829A*^ mRNA@LNPs group. H3K27me3 levels of *KDM6A*^*S829A*^ mRNA@LNPs group were reduced, while Caspase-1 and GSDMD levels were increased compared to vehicle group (Fig. 6F). These data demonstrate that delivering *KDM6A*^*S829A*^ mRNA@LNPs significantly inhibits tumor growth and trigger pyroptosis of HNSCC cells in vivo.

### Delivering *KDM6A*^*S829A*^ mRNA@LNPs promotes anti-tumor immunity in HNSCC

**Fig. 7:**
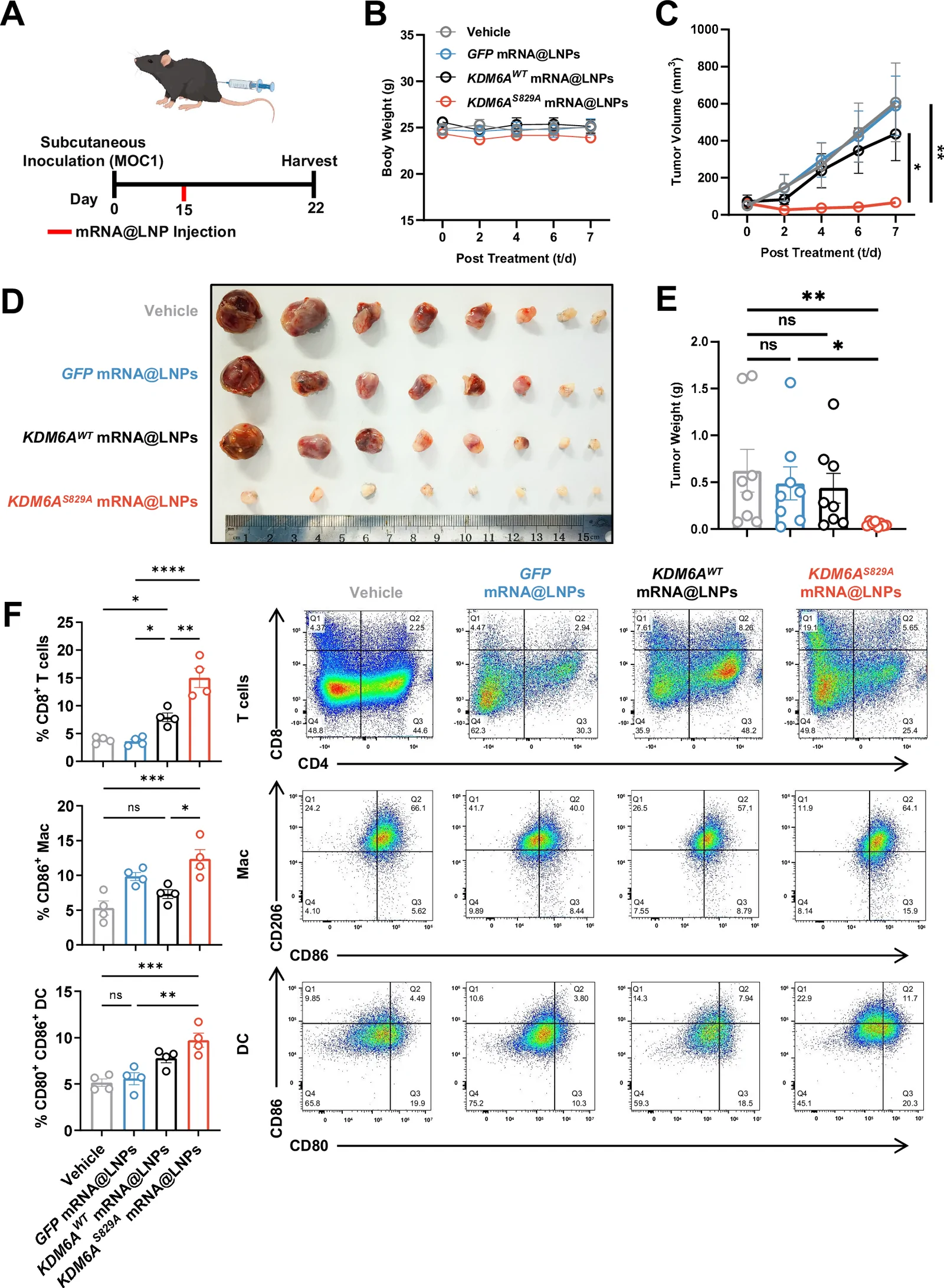
Delivering *KDM6A*^*S829A*^ mRNA@LNPs promotes anti-tumor immunity in HNSCC. **A** Schematic diagram of MOC1 subcutaneous xenograft model illustrates mRNA@LNPs injection (0.5 μg/g). **B** Quantification of body weight the mice at indicated days after treatment (*n* = 8 per group). **C** Quantification of tumor volume of the mice at indicated days after treatment (*n* = 8 per group). **D** Images of tumors in the MOC1 subcutaneous model treated with vehicle, *GFP* mRNA@LNPs, *KDM6A*^*WT*^ mRNA@LNPs and *KDM6A*^*S829A*^ mRNA@LNPs. **E** Tumor weight after the mice were treated with vehicle, *GFP* mRNA@LNPs, *KDM6A*^*WT*^ mRNA@LNPs and *KDM6A*^*S829A*^ mRNA@LNPs. **F** FACS analysis to indicate percentage of CD8^+^ T Cells, CD86^+^ macrophages and CD80^+^ CD86^+^ DC cells in MOC1 tumor tissues under indicated treatments (*n* = 4 per group). Ns, no significance; *p < 0.1, **p < 0.01, ***p < 0.001, ****p < 0.0001.

There has been reported that delivering KDM6A mRNA via LNPs can inhibit the metastasis of bladder cancer [34], supporting the feasibility of KDM6A mRNA-based restoration as an in vivo therapeutic strategy. Based on our mechanistic findings that S829 phosphorylation modulates the tumor-suppressive function of KDM6A, we next evaluated the anti-tumor effects of LNPs containing *GFP* (as a control), *KDM6A*^*WT*^ or *KDM6A*^*S829A*^ mRNA in vivo using a MOC1 tumor model in C57BL/6 mice (Fig. 7A). After the average tumor volume reached 50 mm^3^, mice were treated with PBS or mRNA@LNPs. There was no significant change in body weight among four groups (Fig. 7B). Compared with vehicle or *GFP* mRNA@LNPs group, the injection of *KDM6A*^*S829A*^ mRNA@LNPs significantly inhibited tumor growth, while limited anti-tumor effect was observed in *KDM6A*^*WT*^ @LNPs group (Fig. 7C–E). These results demonstrated that KDM6A^S829A^ has a stronger anti-tumor effect compared with the wild type of KDM6A. Meanwhile, no significant difference was observed in the tumor growth between vehicle group and *GFP* mRNA@LNPs group, indicating that mRNA@LNPs does not account for the therapeutic effect. Furthermore, to evaluate the immune microenvironment, we collected the xenograft tissues and performed FACS examination. While an increased CD8^+^T cells infiltration was observed in the xenograft tissues treated with *KDM6A*^*WT*^ @LNPs, more significant infiltrations of CD8^+^ T cells, CD86^+^ macrophages and CD80^+^ CD86^+^ DC were observed in the xenograft tissues treated with *KDM6A*^*S829A*^ mRNA@LNPs (Fig. 7F), indicating the more activated anti-tumor immune microenvironment after *KDM6A*^*S829A*^ mRNA@LNPs treatment. Taken together, these data demonstrated that delivery of *KDM6A*^*S829A*^ mRNA@LNPs can more effectively activate anti-tumor immunity compared with *KDM6A*^*WT*^ mRNA@LNPs.

**Fig. 8:**
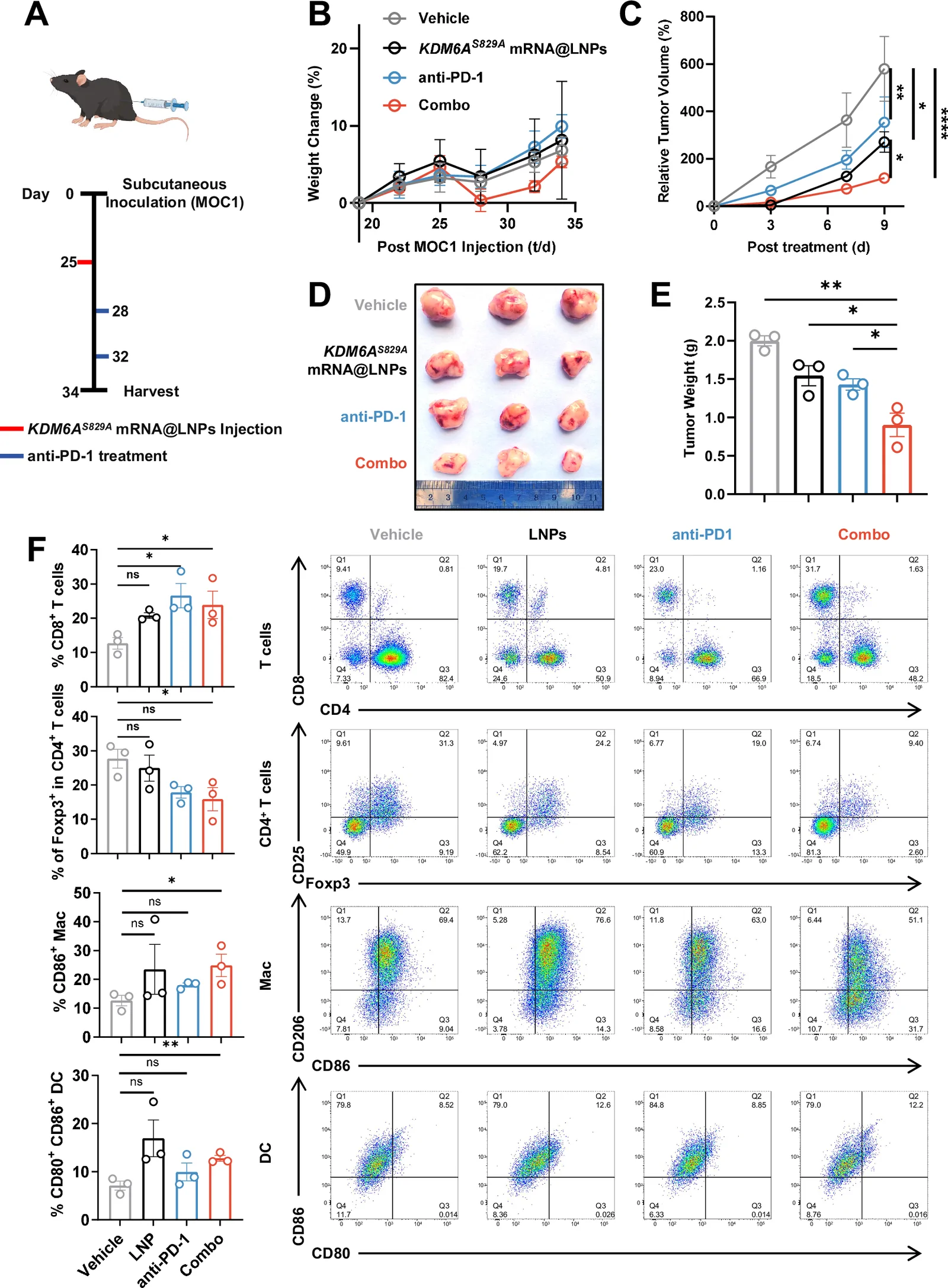
Delivering *KDM6A*^*S829A*^ mRNA@LNPs enhanced PD-1 blockade efficacy in HNSCC. **A** Schematic diagram of MOC1 subcutaneous xenografts model illustrated anti-PD-1 therapy (10 mg/kg) and *KDM6A*^*S829A*^ mRNA@LNPs injection (0.5 μg/g). **B** Quantification of weight change of the mice at indicated days after treatment (n = 3 per group). **C** Quantification of relative tumor volume (RTV) of the mice at indicated days after treatment (*n* = 3 per group). **D** Images of tumors in the MOC1 subcutaneous model treated with vehicle, *KDM6A*^*S829A*^ mRNA@LNPs, PD-1 antibody or combination. **E** Tumor weight after the mice were treated with vehicle, *KDM6A*^*S829A*^ mRNA@LNPs, PD-1 antibody or combination. **F** FACS analysis to indicate percentage of CD8^+^ T Cells, Foxp3^+^ CD4^+^ T cells, CD86^+^ macrophages, and CD80^+^ CD86^+^ DC cells in MOC1 tumor tissues under indicated treatments (*n* = 3 per group). Ns, no significance; *p < 0.1, **p < 0.01, ***p < 0.001, ****p < 0.0001.

To examine whether delivery of *KDM6A*^*S829A*^ mRNA@LNPs synergistically inhibits tumor growth with PD-1 antibody, we established MOC1 xenografts in C57BL/6 mice. After the average tumor volume had reached at 200 mm^3^, mice were treated with PBS, *KDM6A*^*S829A*^ mRNA@LNPs, anti-PD-1 or combination strategy (Fig. 8A). Compared with the other three groups, the combination of *KDM6A*^*S829A*^ mRNA@LNPs and anti-PD-1 significantly reduced tumor burden (Fig. 8C) without influence on body weight (Fig. 8B). Tumors in the *KDM6A*^*S829A*^ mRNA@LNPs or anti-PD-1 group were smaller than the vehicle group, and the tumors from the combination group were the smallest among the four groups (Fig. 8D, E). To detect whether the combination strategy increased the anti-tumor immune cell infiltration, we collected the xenograft tissues and performed FACS examination. Compared with the vehicle group, the combination strategy significantly increased the number of CD8^+^ T cells, CD86^+^ macrophages and CD80^+^ CD86^+^ DC, while the CD25^+^ Foxp3^+^ CD4^+^ T cells were significantly decreased (Fig. 8F). Taken together, these data demonstrated that delivery of *KDM6A*^*S829A*^ mRNA@LNPs combined with anti-PD-1 can suppress tumor growth synergistically.

## Discussions

In this study, we aim to identify actionable epigenetic mechanisms that stimulate pyroptosis to enhance the responsiveness of PD-1 blockade in HNSCC. We identified Ser829 phosphorylation of KDM6A as a pivotal regulator of pyroptosis signaling in HNSCC. Inhibiting KDM6A-pSer829 promoted NLRP3 and GSDMD expression via erasing H3K27me3 and triggered pyroptosis of tumor cells. Delivery of *KDM6A*^*S829A*^ mRNA via LNPs elicited robust anti-tumor effects. Importantly, combination treatment with PD-1 blockades and *KDM6A*^*S829A*^ mRNA@LNPs exhibited synergistic effects, resulting in superior anti-tumor efficacy.

Histone methylation, particularly H3K27me3, is a pivotal epigenetic modification that regulates gene silencing and is implicated in tumorigenesis [35, 36]. As the primary demethylase for H3K27me3, KDM6A stabilizes chromatin structure and suppresses genomic instability [37]. Mutations or downregulation of KDM6A lead to H3K27me3 accumulation, driving oncogenesis in cancers such as bladder and renal cell carcinoma [38]. Notably, many tumors, including HNSCC, retain wild-type KDM6A without altered expression, suggesting post-translational modifications (PTMs) may regulate its activity. Therefore, investigation into the PTMs of KDM6A is needed to provide therapeutic targets to restore or enhance KDM6A’s tumor-suppressive functions. We revealed that the phosphorylation of KDM6A at Ser829 emerges as a critical post-translational modification that governs its dual role as an epigenetic regulator and immune modulator. By abolishing this phosphorylation, KDM6A^S829A^ decreased H3K27me3 levels, enabling the removal of repressive chromatin marks.

The therapeutic potential of *KDM6A*^*S829A*^ mRNA lies in its ability to restore pyroptosis, a highly immunogenic form of cell death, in tumors resistant to PD-1 blockade. By targeting the phosphorylation of KDM6A at Ser829, *KDM6A*^*S829A*^ mRNA effectively reverses the epigenetic silencing of pyroptosis-related genes (e.g., *NLRP3*, *GSDMD*), thereby reinstating a pro-inflammatory tumor microenvironment. This reactivation of pyroptosis not only directly reduces tumor cell viability but also releases damage-associated molecular patterns (DAMPs) and cytokines, such as IL-1β and IL-18, which recruit and activate immune cells, including dendritic cells and cytotoxic T lymphocytes [39]. This dual mechanism enhances the immunogenicity of “cold” tumors, sensitizing them to PD-1 blockade. Furthermore, the use of mRNA-based therapy offers advantages such as transient yet potent gene expression, minimizing off-target effects and enabling precise temporal control. Our findings underscore the translational value of combining *KDM6A*^*S829A*^ mRNA with ICB, providing a promising strategy to overcome resistance and improve outcomes in patients with solid tumors. This approach not only addresses the limitations of current ICB therapies but also opens new avenues for leveraging epigenetic regulation to enhance cancer immunotherapy.

While our study demonstrated the therapeutic potential of *KDM6A*^*S829A*^ mRNA@LNPs, several limitations warrant consideration. First, although KDM6A is a potential factor to predict the effectiveness of anti-PD-1 therapy, and higher KDM6A expression positively correlates with pyroptosis-related genes in many types of tumors, we only examined its anti-tumor effect in HNSCC cells. Further examinations on more types of tumors should be performed to clarify cellular responses. Second, while the synergistic anti-tumor effect was observed in xenograft models treated with anti-PD-1 and *KDM6A*^*S829A*^ mRNA@LNPs, the relatively small sample size may introduce statistical uncertainty in rare subtypes. And the absence of a KDM6A^WT^ control prevents direct comparison of the synergetic effects between KDM6A^WT^ and KDM6A^S829A^. Third, changes in the expression of *NLRP3* and *GSDMD* mRNA were modest in cells expressing KDM6A^S829A^. Given the potentially low transfection efficiency of plasmid vectors, we employed a more efficient expression vector (LNPs) to deliver *KDM6A*^*S829A*^ mRNA, and the results showed that the fold-change in *NLRP3* and *GSDMD* mRNA levels increased by ~2-fold, indicating that LNPs could effectively induce *GSDMD* and *NLRP3* mRNA expression compared with plasmid vector. Meanwhile, previous studies have suggested that transcriptional regulation may moderately influence pyroptosis-related genes [40]. This aligns with our findings that KDM6A^S829A^ can drive pyroptotic effects. Addressing these limitations will refine therapeutic precision without diminishing the immediate implications of our work.

In summary, our findings reveal that KDM6A phosphorylation is a druggable epigenetic-immune checkpoint, and its targeting via LNPs represents a viable strategy to overcome PD-1 resistance in HNSCC.

## Supplementary information

Fig. S1

Fig. S2

Fig. S3

Fig. S4

Fig. S5

Fig. S6

Fig. S7

Fig. S8

Fig. S9

Table S1

Supplementary Materials

Uncropped Gels and Blots image

Supplementary File

## Data Availability

The raw data generated in this study are publicly available in the Genome Sequence Archive (HRA007194 and HRA007193). The data supporting the findings of this study are available from the corresponding author upon reasonable request.

## References

[1] Burtness B, Harrington KJ, Greil R, Soulières D, Tahara M, de Castro G, et al. Pembrolizumab alone or with chemotherapy versus cetuximab with chemotherapy for recurrent or metastatic squamous cell carcinoma of the head and neck (KEYNOTE-048): a randomised, open-label, phase 3 study. Lancet. 2019;394:1915–28. 10.1016/S0140-6736(19)32591-7 31679945

[2] Finn RS, Qin S, Ikeda M, Galle PR, Ducreux M, Kim T-Y, et al. Atezolizumab plus bevacizumab in unresectable hepatocellular carcinoma. N Engl J Med. 2020;382:1894–905. 10.1056/NEJMoa1915745 32402160

[3] Motzer RJ, Tannir NM, McDermott DF, Arén Frontera O, Melichar B, Choueiri TK, et al. Nivolumab plus Ipilimumab versus sunitinib in advanced renal-cell carcinoma. N Engl J Med. 2018;378:1277–90. 10.1056/NEJMoa1712126 PMC5972549 29562145

[4] Reck M, Rodríguez-Abreu D, Robinson AG, Hui R, Csőszi T, Fülöp A, et al. Pembrolizumab versus chemotherapy for PD-L1–positive non–small-cell lung cancer. N Engl J Med. 2016;375:1823–33. 10.1056/NEJMoa1606774 27718847

[5] Wolchok JD, Chiarion-Sileni V, Gonzalez R, Rutkowski P, Grob J-J, Cowey CL, et al. Overall survival with combined nivolumab and ipilimumab in advanced melanoma. N Engl J Med. 2017;377:1345–56. 10.1056/NEJMoa1709684 PMC5706778 28889792

[6] Jiang P, Gu S, Pan D, Fu J, Sahu A, Hu X, et al. Signatures of T cell dysfunction and exclusion predict cancer immunotherapy response. Nat Med. 2018;24:1550–8. 10.1038/s41591-018-0136-1 PMC6487502 30127393

[7] Hegde PS, Chen DS. Top 10 challenges in cancer immunotherapy. Immunity. 2020;52:17–35. 10.1016/j.immuni.2019.12.011 31940268

[8] Elias EE, Lyons B, Muruve DA. Gasdermins and pyroptosis in the kidney. Nat Rev Nephrol. 2023;19:337–50. 10.1038/s41581-022-00662-0 36596918

[9] Zhao C, Zheng T, Wang R, Lin X, Hu Z, Zhao Z, et al. Synergistically augmenting cancer immunotherapy by physical manipulation of pyroptosis induction. Phenomics. 2024;4:298–312. 10.1007/s43657-023-00140-y PMC11466912 39398428

[10] Li F, Zhang X-Q, Ho W, Tang M, Li Z, Bu L, et al. mRNA lipid nanoparticle-mediated pyroptosis sensitizes immunologically cold tumors to checkpoint immunotherapy. Nat Commun. 2023;14:4233. 10.1038/s41467-023-39938-9 PMC10349854 37454146

[11] Yu D, Wang S, Wang J, Zhang K, Niu Z, Lin N. EZH2–STAT3 signaling pathway regulates GSDMD-mediated pyroptosis in glioblastoma. Cell Death Discov. 2024;10:341. 10.1038/s41420-024-02105-0 PMC11284224 39069522

[12] Guan X, Liu R, Wang B, Xiong R, Cui L, Liao Y, et al. Inhibition of HDAC2 sensitises antitumour therapy by promoting NLRP3/GSDMD-mediated pyroptosis in colorectal cancer. Clin Translat Med. 2024;14:e1692. 10.1002/ctm2.1692 PMC11131357 38804602

[13] Yang X, Xu X, Chen J, Wang Q, Wang G, Ai X, et al. Zoledronic acid regulates the synthesis and secretion of IL-1β through Histone methylation in macrophages. Cell Death Disc. 2020;6:47. 10.1038/s41420-020-0273-4 PMC7289826 32566254

[14] Boutron I, Percie du Sert N, Hurst V, Ahluwalia A, Alam S, Avey MT, et al. The ARRIVE guidelines 2.0: Updated guidelines for reporting animal research. PLOS Biol. 2020;18:3617–24. 10.1111/bph.15193 PMC7393194 32662519

[15] Ji Y, Zhang Z, Zhao X, Li Z, Hu X, Zhang M, et al. IL-1α facilitates GSH synthesis to counteract oxidative stress in oral squamous cell carcinoma under glucose deprivation. Cancer Lett. 2024;589:216833. 10.1016/j.canlet.2024.216833 38548217

[16] Zhou Y, Zhou B, Pache L, Chang M, Khodabakhshi AH, Tanaseichuk O, et al. Metascape provides a biologist-oriented resource for the analysis of systems-level datasets. Nat Commun. 2019;10:1523. 10.1038/s41467-019-09234-6 PMC6447622 30944313

[17] Hu X, Ji Y, Zhang M, Li Z, Pan X, Zhang Z, et al. Targeting CXCL8 signaling sensitizes HNSCC to anlotinib by reducing tumor-associated macrophage-derived CLU. J Exper Clin Cancer Res. 2025;44:39. 10.1186/s13046-025-03298-7 PMC11796229 39905539

[18] Li Z, Ji Y, Su Y, Zhou Z, Yang X, Huang Y, et al. “Balloon-like” biomimetic erythrocyte vesicles potentiate photodynamic therapy for inducing immunogenic cell death. Chem Eng J. 2024;500:157406.

[19] Xu X, Chen J, Li Y, Yang X, Wang Q, Wen Y, et al. Targeting epigenetic modulation of cholesterol synthesis as a therapeutic strategy for head and neck squamous cell carcinoma. Cell Death Dis. 2021;12:482. 10.1038/s41419-021-03760-2 PMC8119982 33986254

[20] Wang E, Mounir M, Lucchetta M, Silva TC, Olsen C, Bontempi G, et al. New functionalities in the TCGAbiolinks package for the study and integration of cancer data from GDC and GTEx. PLOS Comput Biol. 2019;15:e1006701. 10.1371/journal.pcbi.1006701 PMC6420023 30835723

[21] Tang Z, Kang B, Li C, Chen T, Zhang Z. GEPIA2: an enhanced web server for large-scale expression profiling and interactive analysis. Nucleic Acids Res. 2019;47:W556–W60. 10.1093/nar/gkz430 PMC6602440 31114875

[22] Liao Y, Savage SR, Dou Y, Shi Z, Yi X, Jiang W, et al. A proteogenomics data-driven knowledge base of human cancer. Cell Syst. 2023;14:777–87.e5. 10.1016/j.cels.2023.07.007 PMC10530292 37619559

[23] Győrffy B. Integrated analysis of public datasets for the discovery and validation of survival-associated genes in solid tumors. The Innovat. 2024;5:100625. 10.1016/j.xinn.2024.100625 PMC11066458 38706955

[24] Szklarczyk D, Kirsch R, Koutrouli M, Nastou K, Mehryary F, Hachilif R, et al. The STRING database in 2023: protein–protein association networks and functional enrichment analyses for any sequenced genome of interest. Nucleic Acids Res. 2023;51:D638–D46. 10.1093/nar/gkac1000 PMC9825434 36370105

[25] Li T, Fan J, Wang B, Traugh N, Chen Q, Liu JS, et al. TIMER: a web server for comprehensive analysis of tumor-infiltrating immune cells. Cancer Res. 2017;77:e108–e10. 10.1158/0008-5472.CAN-17-0307 PMC6042652 29092952

[26] Bhardwaj A, Panepinto MC, Ueberheide B, Neel BG. A mechanism for hypoxia-induced inflammatory cell death in cancer. Nature. 2024;637:470–7. 10.1038/s41586-024-08136-y 39506105

[27] Xiao Y, Zhao C, Tai Y, Li B, Lan T, Lai E, et al. STING mediates hepatocyte pyroptosis in liver fibrosis by epigenetically activating the NLRP3 inflammasome. Redox Biol. 2023;62:102691. 10.1016/j.redox.2023.102691 PMC10106968 37018971

[28] Huang M, Wang Q, Long F, Di Y, Wang J, Zhun Zhu Y, et al. Jmjd3 regulates inflammasome activation and aggravates DSS-induced colitis in mice. FASEB J. 2020;34:4107–19. 10.1096/fj.201902200RR 31971317

[29] Wolf SJ, Audu CO, Moon JY, Joshi AD, Melvin WJ, Barrett EC, et al. Diabetic wound keratinocytes induce macrophage JMJD3-mediated Nlrp3 expression via IL-1R signaling. Diabetes. 2024;73:1462–72. 10.2337/db23-0968 PMC11333374 38869447

[30] Tong L, Song H, Gao Y, Qin D, Wang C, Li Q, et al. Histone demethylase KDM4B epigenetically controls NLRP3 expression to enhance inflammatory responses. EMBO Mol Med. 2026;18:891–915. 10.1038/s44321-026-00373-0 PMC12987974 41703092

[31] Chang S, Yim S, Park H. The cancer driver genes IDH1/2, JARID1C/ KDM5C, and UTX/ KDM6A: crosstalk between histone demethylation and hypoxic reprogramming in cancer metabolism. Exp Mol Med. 2019;51:1–17. 10.1038/s12276-019-0230-6 PMC6586683 31221981

[32] Barbie DA, Tamayo P, Boehm JS, Kim SY, Moody SE, Dunn IF, et al. Systematic RNA interference reveals that oncogenic KRAS-driven cancers require TBK1. Nature. 2009;462:108–12. 10.1038/nature08460 PMC2783335 19847166

[33] Xiao Y, Chen J, Zhou H, Zeng X, Ruan Z, Pu Z, et al. Combining p53 mRNA nanotherapy with immune checkpoint blockade reprograms the immune microenvironment for effective cancer therapy. Nat Commun. 2022;13:758. 10.1038/s41467-022-28279-8 PMC8828745 35140208

[34] Kong N, Zhang R, Wu G, Sui X, Wang J, Kim NY, et al. Intravesical delivery of KDM6A-mRNA via mucoadhesive nanoparticles inhibits the metastasis of bladder cancer. Proc Natl Acad Sci USA. 2022;119:e2112696119. 10.1073/pnas.2112696119 PMC8851555 35131941

[35] Bates SE, Longo DL. Epigenetic therapies for cancer. N Engl J Med. 2020;383:650–63. 10.1056/NEJMra1805035 32786190

[36] Ngollo M, Lebert A, Daures M, Judes G, Rifai K, Dubois L, et al. Global analysis of H3K27me3 as an epigenetic marker in prostate cancer progression. BMC Cancer. 2017;17:261. 10.1186/s12885-017-3256-y PMC5388998 28403887

[37] Cottone L, Cribbs AP, Khandelwal G, Wells G, Ligammari L, Philpott M, et al. Inhibition of histone H3K27 demethylases inactivates brachyury (TBXT) and promotes chordoma cell death. Cancer Res. 2020;80:4540–51. 10.1158/0008-5472.CAN-20-1387 PMC7616956 32855205

[38] Shi B, Li W, Song Y, Wang Z, Ju R, Ulman A, et al. UTX condensation underlies its tumour-suppressive activity. Nature. 2021;597:726–31. 10.1038/s41586-021-03903-7 PMC9008583 34526716

[39] Rao Z, Zhu Y, Yang P, Chen Z, Xia Y, Qiao C, et al. Pyroptosis in inflammatory diseases and cancer. Theranostics. 2022;12:4310–29. 10.7150/thno.71086 PMC9169370 35673561

[40] Jin Y-p, Xu B-j, Zhang X-f, Wang X, Wang L, Li L-y, et al. Long non-coding RNA STMN1P2 promotes breast cancer doxorubicin resistance by inhibiting pyroptosis through the hnRNPU-EZH2-TARF6-MALT1-caspase-1 pathway. Acta Pharmacol Sin. 2025;47:419–33. 10.1038/s41401-025-01653-0 PMC12811385 40926024

