# Supplementary material for: Reversing KDM6A phosphorylation-driven epigenetic suppression restores pyroptosis and synergizes with anti-PD-1 therapy in HNSCC
Source: Cell Death Dis. 2026 Aug 18;17(1):966. doi: 10.1038/s41419-026-09196-w (PMC13759508; doi:10.1038/s41419-026-09196-w)
Supplement: Supplementary file 1 [file 41419_2026_9196_MOESM10_ESM.docx]

| Characteristics | Number of patients | pKDM6A expression (*10^6^) | KDM6A expression  (*10^6^) | pKDM6A/  KDM6A | *p*-Value |
| --- | --- | --- | --- | --- | --- |
| Age (Years) |  |  |  |  | 0.066 |
| ≥60 | 66 | 3.46 | 2.79 | 1.34 |  |
| ＜60 | 34 | 3.64 | 3.32 | 1.18 |  |
| Gender |  |  |  |  | 0.135 |
| Male | 55 | 3.44 | 2.85 | 1.35 |  |
| Female | 45 | 3.62 | 3.12 | 1.21 |  |
| Lymph node |  |  |  |  | 0.056 |
| Non-metastasis | 75 | 3.61 | 2.90 | 1.33 |  |
| Metastasis | 25 | 3.25 | 3.20 | 1.16 |  |
| Tumor Size |  |  |  |  | 0.0209 |
| T1 | 20 | 3.65 | 3.44 | 1.10 |  |
| T2 | 30 | 3.38 | 2.86 | 1.32 |  |
| T3/T4 | 50 | 3.56 | 2.85 | 1.34 |  |
| Pathologic stage |  |  |  |  | 0.0063 |
| I | 37 | 3.43 | 3.23 | 1.18 |  |
| II | 54 | 3.57 | 2.90 | 1.31 |  |
| III/IV | 9 | 3.58 | 2.34 | 1.55 |  |
