# Supplementary material for: Reversing KDM6A phosphorylation-driven epigenetic suppression restores pyroptosis and synergizes with anti-PD-1 therapy in HNSCC
Source: Cell Death Dis. 2026 Aug 18;17(1):966. doi: 10.1038/s41419-026-09196-w (PMC13759508; doi:10.1038/s41419-026-09196-w)
Supplement: Supplementary file 2 [file 41419_2026_9196_MOESM12_ESM.docx]

**Figure 2B**

1. **pS829-KDM6A**

1. **KDM6A**

1. **GSDMD and N-GSDMD**

1. **β-Actin**

**Figure2C**

1. **GSDMD and N-GSDMD**

1. **β-Actin**

**Figure3A**

1. **KDM6A**

1. **H3K27me3**

1. **β-actin**

**Figure 4F**

**HN6**

**SCC7**

**MOC1**

1. **p-KDM6A**

1. KDM6A

1. **H3K27me3**

1. **β-actin**

**Figure 4A**

**Figure S3**

1. **pS829-KDM6A**

1. **KDM6A**

1. **β-Actin**
