# Supplementary material for: Reversing KDM6A phosphorylation-driven epigenetic suppression restores pyroptosis and synergizes with anti-PD-1 therapy in HNSCC
Source: Cell Death Dis. 2026 Aug 18;17(1):966. doi: 10.1038/s41419-026-09196-w (PMC13759508; doi:10.1038/s41419-026-09196-w)
Supplement: Supplementary file 3 [file 41419_2026_9196_MOESM11_ESM.doc]

**Key Resources Table:**

| **Antibodies** | **Source** | **Cat** | **Applications** |
| --- | --- | --- | --- |
| Anti-KDM6A | CST | 33510 | immunoblot (1:1000), IHC (1:200) |
| Anti-KDM6A-pSer829 | Genescript | N/A | immunoblot (1:1000) |
| Anti-H3K27Me3 | Abclonal | A2363 | IHC (1:200) |
| Anti-GSDMD | Abclonal | A20728 | IHC (1:200) |
| Anti-GSDMD and Cleaved N terminal | Abmart | PU224937 | immunoblot (1:2000) |
| Anti-Caspase-1 | Sellleck | F0461 | IHC (1:200) |
| Anti-PD-1 | Bio X Cell | BE0146 | *In vivo* (10 mg/kg) |
| Anti-mouse CD11b BUV395 | Thermo | 363-0112-82 | FCM (1:100) |
| Anti-mouse F480 APC | eBioscience | 17-4801-82 | FCM (1:100) |
| Anti-mouse CD86 BV421 | BioLegend | 105032 | FCM (1:100) |
| Anti-mouse CD206 PE | BioLegend | 141706 | FCM (1:100) |
| Anti-mouse CD11c PE-Cy7 | BioLegend | 117318 | FCM (1:100) |
| Anti-mouse MHC-II BV786 | Thermo | 417-5321-82 | FCM (1:100) |
| Anti-mouse CD80 FITC | BD | 561954 | FCM (1:100) |
| Anti-mouse CD45 BV510 | BioLegend | 103138 | FCM (1:100) |
| Anti-mouse CD3 PE | BioLegend | 100308 | FCM (1:100) |
| Anti-mouse PD1 BV421 | BioLegend | 135218 | FCM (1:100) |
| Anti-mouse CD8 PE-Cy7 | eBioscience | 25-0081-82 | FCM (1:100) |
| Anti-mouse CD4 FITC | eBioscience | 11-0042-85 | FCM (1:100) |
| Anti-mouse CD25 APC | eBioscience | 17-0251-82 | FCM (1:100) |
| Anti-mouse Foxp3 PE | eBioscience | 12-5773-82 | FCM (1:100) |
| Anti-mouse CD45 FITC | eBioscience | 11-0451-85 | FCM (1:100) |
| Anti-mouse CD4 BV421 | BioLegend | 100544 | FCM (1:100) |

| **Experimental animals** | **Source** | **Cat** |
| --- | --- | --- |
| BALB/c nude mice | Shanghai Laboratory Animal Center | N/A |
| C57BL/6 mice | Shanghai Laboratory Animal Center | N/A |

| **Critical commercial kits** | **Source** | **Cat** |
| --- | --- | --- |
| Annexin V-FITC Apoptosis Detection Kit | Beyotime | C1062 |
| LDH Release Assay Kit | Beyotime | C0019 |
| Enzymatic Chromatin IP Kit | CST | 9003 |
| Lipofectamine 3000 reagent | Thermo Fisher | L3000015 |
| BCA Protein Assay Kit | Thermo Fisher | 23227 |
| CCK-8 Detection Kit | Cellcook | CT01C |
| TRIzol Reagent | Takara | 9108 |
| PrimerScript RT reagent Kit | Takara | 6215 |
| TB Green Premix Ex Taq reagent kit | Takara | RR820 |
| Fixable Viability Dye eF780 | eBioscience | 65-0865-14 |
| Intracellular Fix/Perm Buffer Set | Thermo | 88-8824-00 |

| **Reagents and consumables** | **Source** | **Cat** |
| --- | --- | --- |
| Fetal Bovine Serum | TBD Science | TBD15HT |
| DMEM | BasalMedia | L110KJ |
| IMDM | BasalMedia | L610KJ |
| Ham’s F-12 | BasalMedia | L410KJ |
| Insulin | Selleck | S6955 |
| Hydrocortisone | Selleck | S1696 |
| Recombinant Mouse EGF | AnWei-sci | AW-RM01-001 |
| 0.25%Trypsin-EDTA(1X) | NCM Biotech | C100C1 |
| Penicillin-Streptomycin | TBD Science | PS2004HY |
| RIPA Lysis Buffer | NCM Biotech | WB3100 |
| Skim Milk | YEASEN | 36120ES76 |
| Tris-MOPS-SDS Runing Buffer Powder | GenScript | M00138 |
| 10X TBST Buffer | Sangon Biotech | C520009-0001 |
| Antibody Diluent | NCM Biotech | WB500D |
| Chemiluminescent HRP Substrate | Millipore | WBKLS0500 |
| 4% Paraformaldehyde | Biosharp | BL539A |
| DAPI Staining Solution | Beyotime | C1005 |
| Microplate | Thermo Fisher | 4346906 |

| **Software and algorithms** | **Source** | **Identifier** |
| --- | --- | --- |
| GEPIA2 | Tang, et al. | http://gepia2.cancer-pku.cn/#index |
| LinkedOmicsKB | Liao, et al. | https://kb.linkedomics.org/ |
| KM Plotter | Balázs Győrffy | https://kmplot.com/ |
| FlowJo 10.5.3 | FlowJo Software | https://www.flowjo.com/ |
| GraphPad Prism 9.4.0 | GraphPad Software | https://www.graphpad.com/scientific-software/prism/ |
| R version 4.1.1 | R Core Team | https://www.r-project.org |
| Image J | National Institutes of Health | https://imagej.nih.gov/ij/ |
| FigDraw | NA | https://www.figdraw.com/ |

| **Primer name** | **Forward sequence** | **Reverse sequence** |
| --- | --- | --- |
| H-B-ACTIN | CATGTACGTTGCTATCCAGGC | CTCCTTAATGTCACGCACGAT |
| H-KDM6A | TTCCTCGGAAGGTGCTATTCA | GAGGCTGGTTGCAGGATTCA |
| H-NLRP3 | GATCTTCGCTGCGATCAACAG | CGTGCATTATCTGAACCCCAC |
| H-GSDMD | GTGTGTCAACCTGTCTATCAAGG | CATGGCATCGTAGAAGTGGAAG |
| M-B-ACTIN | GGCTGTATTCCCCTCCATCG | CCAGTTGGTAACAATGCCATGT |
| M-NLRP3 | ATTACCCGCCCGAGAAAGG | TCGCAGCAAAGATCCACACAG |
| M-GSDMD | CCATCGGCCTTTGAGAAAGTG | ACACATGAATAACGGGGTTTCC |
